# *Clostridium butyricum*-altered lung microbiome is associated with enhanced anti-influenza effects via G-protein-coupled receptor120

**DOI:** 10.1016/j.isci.2025.113502

**Published:** 2025-09-04

**Authors:** Mao Hagihara, Makoto Yamashita, Tadashi Ariyoshi, Ayaka Minemura, Chika Yoshida, Seiya Higashi, Kentaro Oka, Motomichi Takahashi, Akinobu Ota, Akihiro Maenaka, Kenta Iwasaki, Jun Hirai, Yuichi Shibata, Takumi Umemura, Takeshi Mori, Hideo Kato, Nobuhiro Asai, Hiroshige Mikamo

**Affiliations:** 1 Department of Molecular Epidemiology and Biomedical Sciences, Aichi Medical University, Nagakute 480-1195, Japan; 2 Department of Clinical Infectious Diseases, Aichi Medical University, Nagakute 480-1195, Japan; 3 R&D Division, Miyarisan Pharmaceutical Co., Ltd., Saitama 331-0804, Japan; 4 Department of Pharmacy, College of Pharmacy Kinjo Gakuin University, Nagoya 463-8521, Japan; 5 Departments of Kidney Disease and Transplant Immunology, Aichi Medical University, Nagakute 480-1195, Japan; 6 Department of Pharmacy, Mie University Hospital, Tsu, Mie, Japan

**Keywords:** Cell biology, Microbiology

## Abstract

We previously reported that orally administered *Clostridium butyricum* enhances anti-influenza virus effects through the interferon-λ upregulation in mice lungs; however, the precise mechanism remains unclear. Orally administered *C. butyricum* promotes the proliferation of *Bifidobacterium* species in the lung microbiome, and this enhances *C. butyricum* induced anti-influenza effects. Among the *Bifidobacterium* species, *B. longum* effectively enhanced the sensitivity of the lung epithelial cells to long-chain fatty acids through the G-protein-coupled receptor120 upregulation. Oral administration of *C. butyricum* altered long-chain fatty acid metabolism and promoted interferon-λ production through G-protein-coupled receptor120. We hypothesized that these effects enhance anti-influenza virus responses through interferon-λ upregulation via collaboration between long-chain fatty acid metabolism alterations and the lung microbiome moderation. This study identified a gut-lung axis mechanism and provides insights into viral respiratory infection treatment and prophylaxis.

## Highlights

• Butyrate-producing *Clostridium butyricum* attenuates influenza virus pneumonia
• Orally administered *C. butyricum* alters the lung microbiome
• The lung microbiome has important anti-influenza virus effects
• Enhanced GPR120 expression by *C. butyricum* is linked to *Bifidobacterium* proliferation

## Graphical abstract

**Figure:**
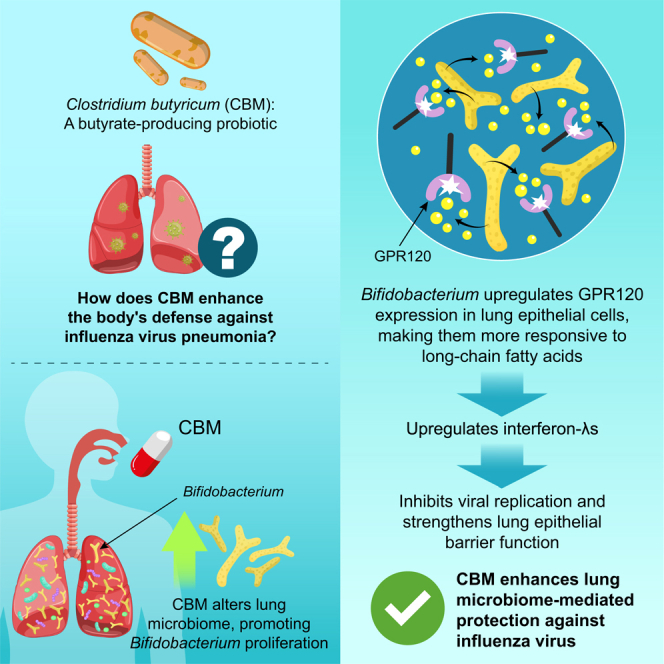

## Introduction

Respiratory viral infections are major public health issues worldwide.^1^^,^^2^ Of these, influenza has been a major respiratory disease for the past century, and its pandemics have profoundly impacted public health.^3^^,^^4^^,^^5^^,^^6^ Additionally, these viral infections account for 3–5 million cases of severe illness annually.^7^ Epidemiological studies have identified influenza virus isolates resistant to antiviral agents, while vaccine effectiveness remains insufficient owing to high genetic variability.^8^^,^^9^^,^^10^ Thus, new and cost-effective measures to efficiently prevent and treat influenza virus infections at a low cost are still required.

The epithelial lining of the respiratory tissue plays a vital role in viral infections, serving both as a point of viral entry and an essential component of antiviral immune response.^11^^,^^12^^,^^13^ Thus, the interaction between pathogenic viruses and host epithelial cells is a critical factor that often determines the outcome of respiratory infections.^14^ However, the protective role of the lung microbiome that interacts with both pathogenic viruses and lung epithelial cells in preventing respiratory viral infections remains unclear. In contrast, the gut microbiome plays an important protective role against these infections by regulating the host immune system,^15^^,^^16^^,^^17^^,^^18^^,^^19^^,^^20^^,^^21^ otherwise referred to as “the gut-lung axis.”

Butyrate-producing bacteria in the gut can affect disease prognosis, with a higher abundance of butyrate-producing bacteria resulting in a lower incidence of hospitalizations and deaths due to infections, including respiratory viral infections.^22^^,^^23^ Orally administered *Clostridium butyricum* MIYAIRI 588 (CBM 588), a gram-positive butyrate-producing anaerobe, enhances resistance to influenza virus infection through the upregulation of type III interferons (IFN-λ) in lung epithelial cells.^24^ Furthermore, CBM 588-induced long-chain fatty acids (LCFAs) promote IFN-λ production in the lungs.

Patients with respiratory viral infections have altered lung and gut microbiomes.^25^^,^^26^^,^^27^ However, it remains unclear whether the alterations in the lung microbiome, as opposed to the gut microbiome, are a consequence of the disease itself or influence the prognosis of respiratory virus infections.^26^^,^^28^^,^^29^^,^^30^^,^^31^ Additionally, the precise mechanisms by which butyrate-producing bacteria in the gut, such as orally administered CBM 588, protect against the progression of viral respiratory infections are still unknown. Therefore, this study aimed to examine the relationship between the lung microbiome and orally administered CBM 588-induced anti-influenza viral effects.

## Results

### CBM 588 demonstrates anti-viral effects against the influenza virus (H3N2)

**Figure 1:**
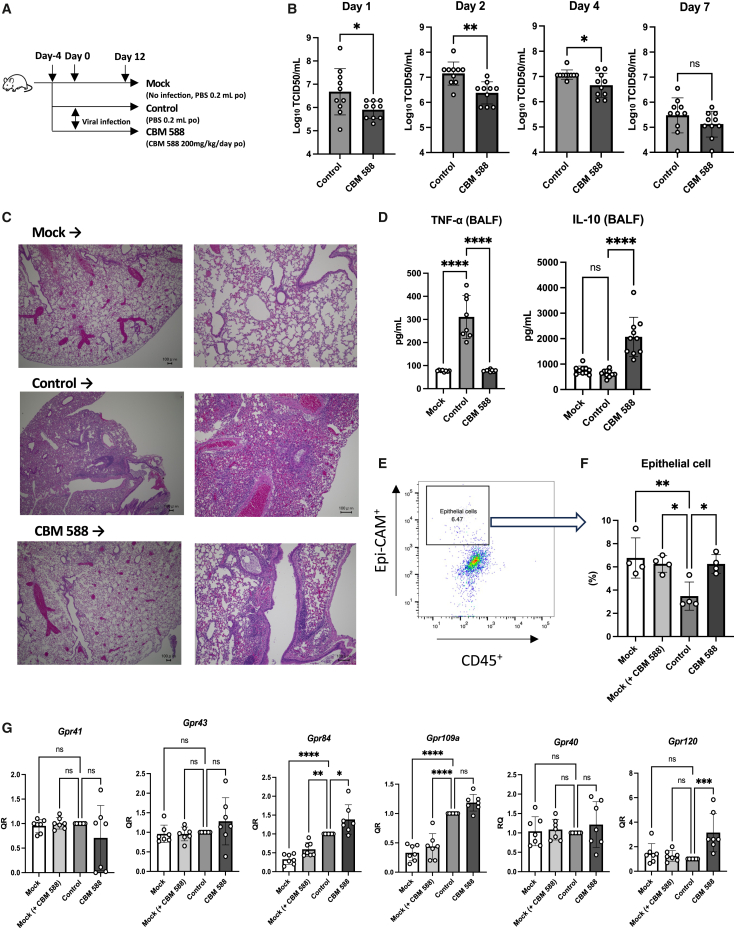
CBM 588 demonstrates anti-influenza virus effects against influenza virus (H3N2) (A) BALB/c mice were administered phosphate-buffered saline (PBS) or *C. butyricum* (CBM 588) for 16 days. The control and CBM 588 groups were infected with the influenza A virus H3N2. Mock, *n* = 10; control, *n* = 10; CBM 588, *n* = 10. (B) Viral titers in the lungs on days 1, 2, 4, and 7 after influenza virus infection. (C) Representative lung histological images on day 2 after influenza virus infection (scale bar, 100 mm at the bottom right). (D) Cytokines in bronchoalveolar lavage (BALF) on day 2 post-infection. (E) Representative flow cytometry plots of lung epithelial cell (CD326^+^/CD45^-^) expression in the isolated cells. Mock, *n* = 4; Mock (+ CBM 588), *n* = 7; control, *n* = 4; and CBM 588, *n* = 4. (F) Percentage of lung epithelial cells in the lungs. (G) Relative expression of G-protein-coupled receptor genes in lung epithelial cells (RQ). Mock, *n* = 7; Mock (+ CBM 588), *n* = 7; control, *n* = 7; CBM 588, *n* = 7. The results are presented as mean ± standard deviation (SD). Each dot represents an individual mouse. Results were considered statistically significant when differences were *p* < 0.05 (∗∗∗∗: *p* ≤ 0.0001, ∗∗∗: *p* ≤ 0.001, ∗∗: *p* ≤ 0.01, ∗: *p* ≤ 0.05; ns indicates not significant). See also Figure S1.

We orally administered CBM 588 to the murine influenza virus (H3N2) infection model, as in our previous *in vivo* study.^24^ In Japan, CBM 588 is used as a probiotic. Hence, we adjusted the dosage of CBM 588 for mice to be the same ratio (CBM 588 dose / day / body weight) as that of humans. When CBM 588 was orally administered day −4 to day 12, CBM 588 reduced influenza virus titer in the virus-infected mouse lungs (Figures 1A and 1B), and the effect showed CBM 588 dose-dependent manner at day 2 (see Figure S1A). Also, 2 days post-infection, CBM 588 attenuated inflammations in the lungs (Figures 1C and 1D), as also noted in our previous study.^24^ The representative cell populations (%) of mononuclear cells in the lungs at day 2, including T cells, monocytes, neutrophils, and macrophages, showed no differences between the control and CBM 588 administration groups (see Figures S1B–S1D). However, CBM 588 administration protected against this reduction in the number of lung epithelial cells (Figures 1E and 1F). Microbiome-produced short-chain fatty acids (SCFAs) contribute to the attenuation of tissue damage owing to the recruitment of immune cells during influenza virus infection in mice and enhance anti-viral effects.^15^ However, CBM 588 administration did not enhance the production of lactate and succinate in the bronchoalveolar lavage fluid (BALF), whereas we did not know whether the other SCFAs (formic acid, propionate, acetate, and butyrate) were altered with CBM 588 as they were under detection limits (see Figure S1E). Additionally, CBM 588 administration did not enhance the production of SCFAs in the fecal sample, whereas we did not know whether the formic acid was altered with CBM 588 as they were under detection limits (see Figure S1F). In contrast, 2 days after virus infection, we processed the murine lungs to separate the epithelial cells with percoll as in previous *in vivo* study,^24^ and conducted cell sorting (the final purity of lung epithelial cells was around 96%). Consequently, CBM 588 enhanced the production of certain LCFAs in lung epithelial cells (see Figure S1G), as observed in our previous *in vivo* study.^24^ Additionally, G-protein-coupled receptor120 (GPR120), one of the main receptors for LCFAs^32^ in murine lung epithelial cells, was significantly upregulated by CBM 588 administration (Figure 1G), whereas CBM 588 administration had minimal effects on other GPCRs, such as GPR40 (another receptor for LCFAs),^32^ as well as GPR41, GPR43, GPR84, and GPR109A, which are receptors for SCFAs.^32^

### Orally administered CBM 588 alters lung and gut microbiome

**Figure 2:**
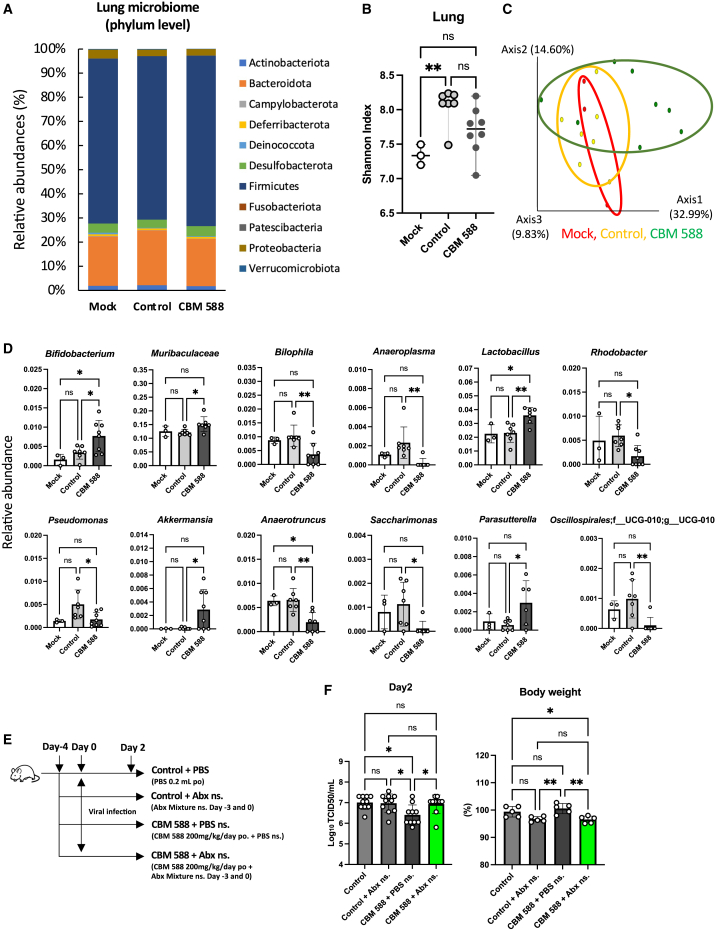
Orally administered CBM 588 alters the lung microbiome to enhance its anti-influenza virus effects (A) Bar graphs depict the mean relative abundance of bacterial families (>1% relative abundance) in the lung microbiome at the phylum level for each group. BALB/c mice received phosphate-buffered saline (PBS) (mock), PBS (control), or *C. butyricum* (CBM 588) and were sacrificed 2 days after virus infection. The control and CBM 588 administration groups were infected with the influenza virus H3N2. Mock, *n* = 4; control, *n* = 7; CB administration group, *n* = 8. (B) Comparison of the Shannon index among different groups. (C) Principal Coordinate Analysis (PCoA) was performed based on weighted UniFrac distances among the mock, control, and CBM 588 administration groups. (D) Abundances of relative species at the genus and species levels (≥0.1%) in lung samples. Data are presented as the mean values of the relative abundance ±SD. (E) BALB/c mice were administered PBS or CBM 588 for 6 days. All groups were infected with influenza A virus H3N2. The control group received oral phosphate buffered saline (PBS). The other group received oral PBS or CBM 588 supplemented with nasal PBS or an antibiotic mixture (Abx). Control, *n* = 10; control (Abx), *n* = 10; CBM 588 + PBS, *n* = 10; CBM 588 + Abx, *n* = 10. (F) Viral titers in the lungs and body weight on day 2, after influenza virus infection. Each dot represents an individual mouse. Results were considered statistically significant when differences were *p* < 0.05 (∗∗*p* < 0.01, ∗*p* < 0.05; ns indicates not significant). See Figure S2.

To elucidate the effect of oral CBM 588 administrations on the microbiome of influenza virus-infected mice, we performed lung and gut microbiome analyses (Figures 2A and S2A). The lung microbiome mainly consists of Firmicutes and Bacteroidetes at the phylum level. Then, viral infection significantly increased α-diversity of the lung microbiome, whereas the CBM 588 administration group showed a decreasing trend (Figure 2B). However, influenza virus infection and CBM 588 administration did not significantly alter α-diversity of the gut microbiome (Figure S2B). There was no significant change in the bacterial community composition (β-diversity) in lung and gut microbiomes between the CBM 588 administration and control groups (Figures 2C and S2C). At the genus level, 12 and 6 bacterial species showed significant differences in their relative abundance (%) in the lung and gut microbiomes, respectively, between the control and CBM 588 administration groups (Figures 2D and S2D). CBM 588 administration resulted in a significant increase in the relative abundances (%), such as *Lactobacillus* and *Bifidobacterium*, compared to those in the control group in the lung microbiome (Figure 2D). However, different bacterial species in the gut microbiome altered their relative abundances with that of the lung microbiome.

### Lung microbiome is important for CBM 588-induced anti-influenza virus effects

To determine the importance of the lung microbiome in influenza virus infection resistance, BALF prepared from mock mice was nasally administered to antibiotic mixture (Abx)-treated and influenza virus-infected mice with normal lung microbiomes (see Figure S2E). Then, the Abx solution was administered to the mice to cause lung microbiome dysbiosis prior to lung microbiome transplantation. Consequently, mice that received lung microbiome transplantation (Abx + BALF group) showed significantly lower influenza virus titers in the lungs than those that received phosphate-buffered saline (PBS) nasally (Abx +PBS) (see Figure S2F). Additionally, to determine the importance of the lung microbiome in the antiviral effects induced by CBM 588, CBM 588 was administered orally along with Abx solution or PBS nasally to influenza virus-infected mice (Figure 2E). Consequently, the dysbiosis of the lung microbiome due to influenza virus infection and antibiotic exposure reduced host influenza virus infection resistance (Figure 2F). Notably, mice that received oral CBM 588 and nasal Abx showed higher viral titers than mice that received oral CBM 588 and nasal PBS (Figure 2F). In contrast, orally administered CBM 588 mice treated with Abx solution or PBS showed CBM 588-induced anti-influenza virus effects (see Figures S2G and S2H). These results revealed that CBM 588-induced lung microbiome alterations play a critical role in mediating its antiviral effects against the influenza virus.

### GPR120 is crucial for CBM 588-induced anti-influenza virus effects

**Figure 3:**
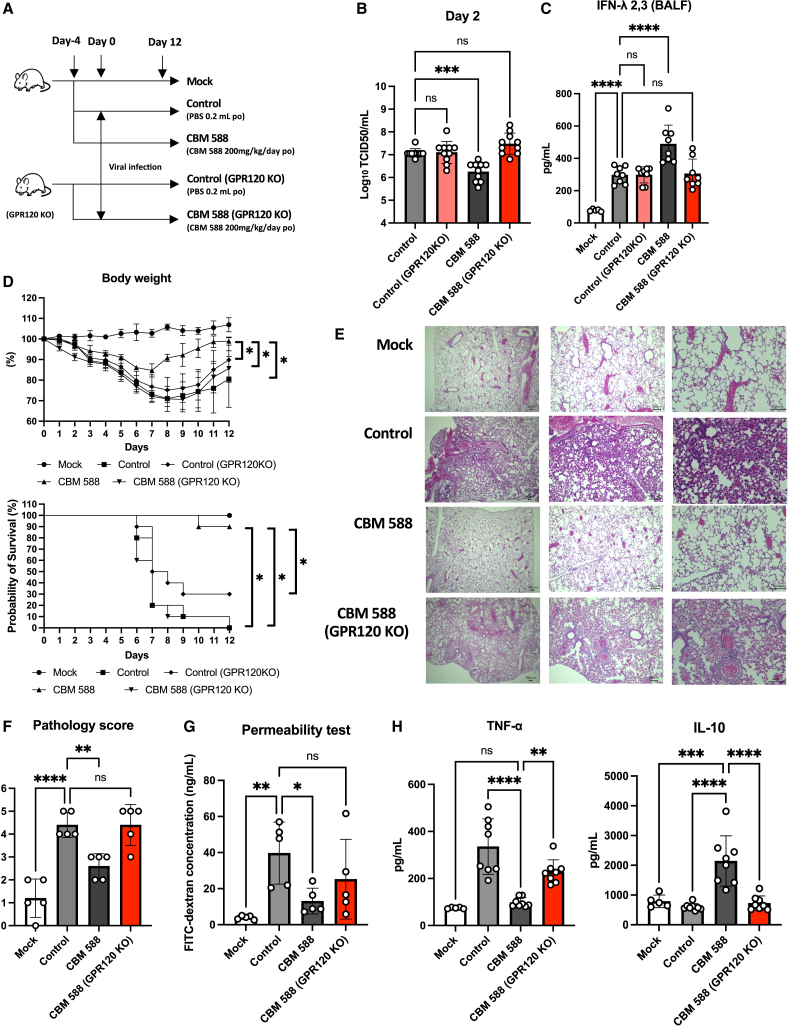
GPR120 plays an important role in CBM 588-induced anti-influenza virus effects (A) BALB/c mice (wild-type or GPR120 KO) received phosphate-buffered saline (PBS) or oral *C. butyricum* (CBM 588) administrations for 16 days. All groups were infected with the influenza A virus H3N2, except for the mock group. Mock, *n* = 8; control, *n* = 8; control (GPR120 KO), *n* = 8; CBM 588, *n* = 8; CBM588 (GPR120 KO), *n* = 8. (B–D) Viral titer in the lungs on day 2 after influenza virus infection (B), IFN-λ2,3 concentration in bronchoalveolar lavage (BALF) (C), body weights, and survival rates (D). (E) Representative lung histological images on day 2 after influenza virus infection (scale bar, 100 mm at the bottom right). (F and G) Pathology scores (F) and lung permeability test results (G) on day 2 post-infection. Mock, *n* = 5; control, *n* = 5; CBM 588, *n* = 5; CBM588 (GPR120 KO), *n* = 5. (H) Cytokines in BALF 2 days post-infection. The results are presented as mean ± standard deviation (SD). Each dot represents an individual mouse. Results were considered statistically significant when differences were *p* < 0.05 (∗∗∗∗: *p* ≤ 0.0001, ∗∗∗: *p* ≤ 0.001, ∗∗: *p* ≤ 0.01, ∗: *p* ≤ 0.05; ns indicates not significant). See also Figure S3.

To understand the involvement of GPR120 in CBM 588-induced anti-influenza virus effects through IFN-λ upregulation,^24^ we conducted an *in vivo* study in GPR120 gene deficient (whole genome *Gpr120*^−/−^) BALB/c mice (Figure 3A). Consequently, in absence of orally administered CBM 588, GPR120 KO mice (Control (GPR120 KO) group) showed comparable results on viral burden in the lung tissue, IFN-λ2,3 production level, body weight loss due to virus infection, and survival ratio with wild type mice (Control group) (Figures 3B–3D). At 2 days post-infection, GPR120 KO mice that received CBM 588 (CBM 588 (GPR120 KO) group) showed significantly higher virus titer in the lungs and significantly lower IFN-λ2,3 expression level in BALF, compared with the wild-type BALB/c mice that received the same CBM 588 dose (CBM 588 group) (Figures 3B and 3C). Moreover, weight loss in the CBM 588 (GPR120 KO) group was more pronounced and had a significantly lower survival rate than that of the CBM 588 group (Figure 3D). The CBM 588 (GPR120 KO) group showed impaired defense abilities in the lungs, such as the downregulation of MUC5AC expression and tight junction proteins (TJs) gene expression, compared to the CBM 588 group (see Figures S3A and S3B). The CBM 588 (GPR120 KO) group showed a significantly higher lung pathology score and lung permeability at day 2 (Figures 3E–3G). Also, 2 days post-infection, the expression of pro-inflammatory cytokines in BALF, such as tumor necrosis factor-α (TNF-α), was significantly higher in the CBM 588 (GPR120 KO) group than in that of the CBM 588 group, whereas the expression of anti-inflammatory cytokine IL-10 was significantly reduced (Figure 3H). Orally administered CBM 588 is known to promote IFN-λ production in the lungs through IFN regulatory factor (IRF)-7 activation.^24^ However, the CBM 588 (GPR120 KO) group downregulated *Irf-7* gene expression compared to that of the CBM 588 group (see Figure S3C). In contrast, CBM 588 administered orally to IRF-7KO mice (CBM 588 (IRF-7 KO) group) showed comparable *Gpr120* gene expression levels to those of the CBM 588 group (see Figure S3D).

### CBM 588-induced *Bifidobacterium* species upregulate GPR120 expression

**Figure 4:**
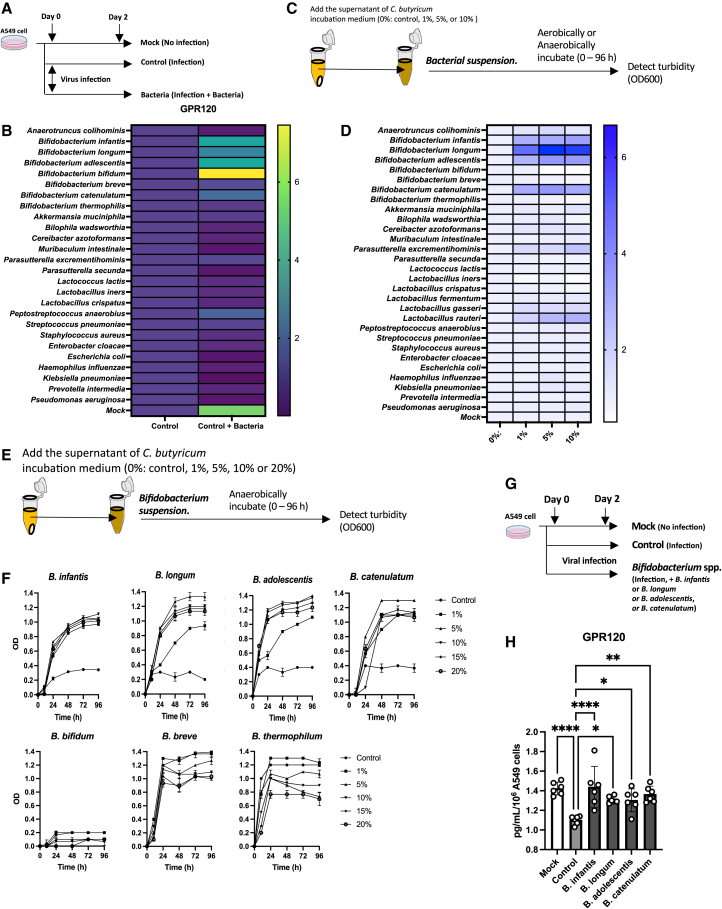
CBM 588-induced *Bifidobacterium* species upregulates GPR120 expression (A) A549 cells were exposed to various bacterial species during influenza A virus H3N2 infection, and incubated anaerobically for 48 h. Mock, *n* = 6; control, *n* = 6; bacteria, *n* = 6. (B) The heatmap represents the rate of GPR120 expression in A549 cells after 48 h of incubation. (A549 cells were exposed to each bacterium during influenza virus infection/A549 cells were not exposed to bacteria during influenza virus infection). (C) The supernatants of *C. butyricum* (CBM 588) incubation medium were added to each bacterial suspension at 0% (control), 1%, 5%, and 10%, and their turbidity was detected within 96 h of incubation under aerobic or anaerobic conditions. (D) The heatmap represents the ratio of bacterial concentrations after 24 h of incubation. The CBM 588 incubation medium supernatant was added to each bacterial suspension at 1%, 5%, and 10% of each bacterial suspension without the supernatant (0%). (E) The supernatants of the CBM 588 incubation medium were added to *Bifidobacterium* species suspension at 0% (control), 1%, 5%, 10%, 15%, and 20%, and the turbidity was detected anaerobically within 96 h of incubation. *n* = 6 for each species. (F) The turbidity *of Bifidobacterium* species, including medium within 0–96 h incubation. (G) A549 cells were exposed to each *Bifidobacterium* species for 48 h under influenza virus H3N2 infection, with the exception of the mock group. Mock, *n* = 6; control, *n* = 6; *Bifidobacterium* species, *n* = 6. (H) Protein expressions of GPR120 in A549 cells. The results are presented as mean ± standard deviation (SD). Each dot represents an individual mouse. Results were considered statistically significant when differences were *p* < 0.05 (∗∗∗∗: *p* ≤ 0.0001, ∗∗∗: *p* ≤ 0.001, ∗∗: *p* ≤ 0.01, ∗: *p* ≤ 0.05; ns indicates not significant). See also Figure S4.

Given that the gene expression of GPCRs in germ-free mice is reduced,^33^ we expected that alterations in the lung microbiome composition induced by CBM 588 administration would affect GPR120 expression in lung epithelial cells. To investigate the effects of various bacterial species on the *Gpr120* gene expression levels (Figure 4A) in A549 cell lines under influenza virus infection, except the mock group (no-virus infection), we exposed 30 different isolates, picked up based on our lung microbiome study results, to cultured human lung epithelial cells. Consequently, we found *Bifidobacterium* species can enhance *Gpr120* gene expression (Figure 4B), and some butyrate-producing bacteria, including CBM 588, can also induced *Gpr120* gene expression (Figure S4A). GPR40 expression levels were not altered by exposures of *Bifidobacterium* species (see Figure S4B). To elucidate how orally administered CBM 588 alters the lung microbiome, we conducted an additional *in vitro* study (Figures 4C and 4D). Then, the culture supernatant of CBM 588 promoted proliferations of particular *Bifidobacterium* species, such as *B. infantis*, *B. longum*, *B. adolescentis,* and *B. catenulatum* (Figures 4E and 4F), whereases these effects were not observed upon heating the culture supernatants of CBM 588 or preincubating them with proteinase K (see Figures S4C and S4D). Additionally, we demonstrated that *B. infantis*, *B. longum, B. adolescentis,* and *B. catenulatum* significantly upregulated GPR120 protein expression compared to the controls in our *in vitro* studies (Figures 4G and 4H).

### *Bifidobacterium longum* promotes IFN-λ production through GPR120 upregulation

**Figure 5:**
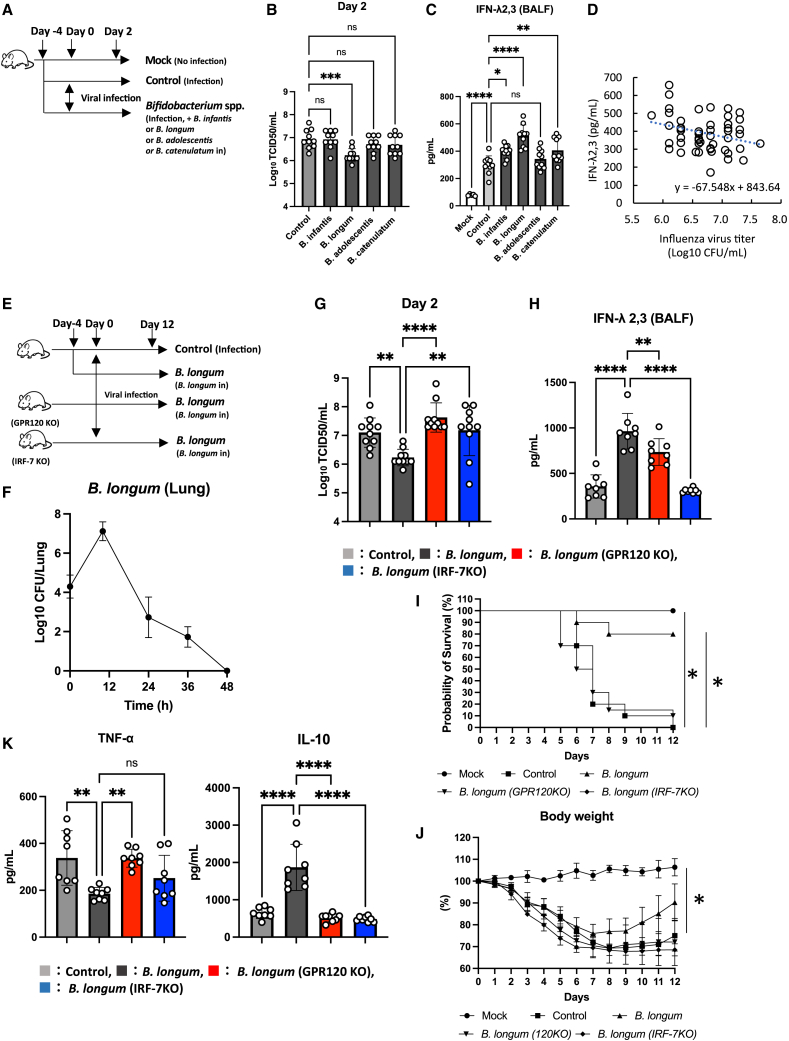
*Bifidobacterium longum* promotes IFN-λ production through GPR120 upregulation (A) BALB/c mice received phosphate-buffered saline (PBS) (mock), PBS (control), or *B. infantis*, *B. longum*, *B. adolescentis,* or *B. catenulatum* administration nasally for 16 days. All groups were exposed to influenza virus H3N2, except for the mock group. Mock, *n* = 5; control, *n* = 10; *Bifidobacterium* species administered group, *n* = 10. (B) Viral titers in the lungs on day 2 after influenza virus infection. (C) INF-λ2,3 concentrations in BALF. (D) Correlation between IFN-λ concentrations in BALF and viral titers in murine lung tissues. (E) Mice received PBS (control) or *B. longum* via the nasal cavity and were sacrificed 2 days after virus infection. All groups were exposed to the influenza virus H3N2. Control, *n* = 10; *B. longum*, *n* = 10; *B. longum* (GPR120 KO), *n* = 10; *B. longum* (IRF-7 KO), *n* = 10. (F) Bacterial concentrations in the lungs after the single nasal *B. longum* administration. (G) Viral titer in the lungs on day 2 after influenza virus infection. (H) INF-λ2,3 concentrations in bronchoalveolar lavage (BALF). (I–K) Survival rates (I), body weight (J), and cytokine levels in the BALF on day 2 post-infection (K). The results are presented as mean ± standard deviation (SD). Each dot represents an individual mouse. Results were considered statistically significant when differences were *p* < 0.05 (∗∗∗∗: *p* ≤ 0.0001, ∗∗∗: *p* ≤ 0.001, ∗∗: *p* ≤ 0.01, ∗: *p* ≤ 0.05; ns indicates not significant). See also Figure S5.

To know the impacts of the *Bifidobacterium* species on IFN-λ production through GPR120, we compared the IFN-λ expression levels in the wild type (control) and GPR120 KO (*gpr120*^−/−^) human epithelial cells with IRF-7 KO (*irf*-7^−/−^) human epithelial cells after exposure to *B. infantis*, *B. longum, B. adolescentis,* and *B. catenulatum* (see Figures S5A and S5B), as IRF-7 is a known upregulator of CBM 588-induced IFN-λ.^24^ *B. infantis*, *B. adolescentis,* and *B. catenulatum* increased IFN-λ production. However, GPR120 KO cells showed significant down-regulations of IFN-λ expression levels, as observed in IRF-7 KO cells (see Figure S5C). Additionally, the nasal administration of *B. infantis*, *B. longum*, *B. adolescenti*s, and *B. catenulatum* enhanced resistance to influenza virus infection (Figures 5A–5C). Then, influenza virus titers in murine lung tissues showed a negative correlation with IFN-λ concentrations in BALF (Figure 5D). Notably, the *B. longum* nasal administration group showed the highest IFN-λ2,3 expression level and the lowest viral titer in the lungs compared to other *Bifidobacterium* species (Figures 5B and 5C). To determine the effects of GPR120 and IRF-7 on nasally administered *B. longum*-induced anti-influenza virus effects, we conducted *in vivo* experiments (Figure 5E). Nasal administration of *B. longum* resulted in its presence in the lungs for up to 36 h (Figure 5F), with significantly lower virus titer in the lungs and higher IFN-λ2,3 expression level on day 2 post-infection, compared with the control (Figures 5G and 5H). Weight loss in mice was attenuated, and the survival rate of the *B. longum* administration group was higher than that in the control group (Figures 5I and 5J). Two days post-infection, the expression of TNF-α in BALF was significantly lower in the *B. longum* administration group than that of controls (Figure 5K). However, a significant reduction in *B. longum*-induced anti-influenza virus effects, along with the down-regulation of IFN-λ2,3 expression levels in the lungs, was observed in both the GPR120 KO and IRF-7 KO mice (Figures 5G and 5H). Similarly, these KO mice showed exaggerated symptoms of viral infection (Figures 5I–5K).

### *B. longum* synergistically enhances LCFAs-induced anti-influenza virus effects via GPR120

**Figure 6:**
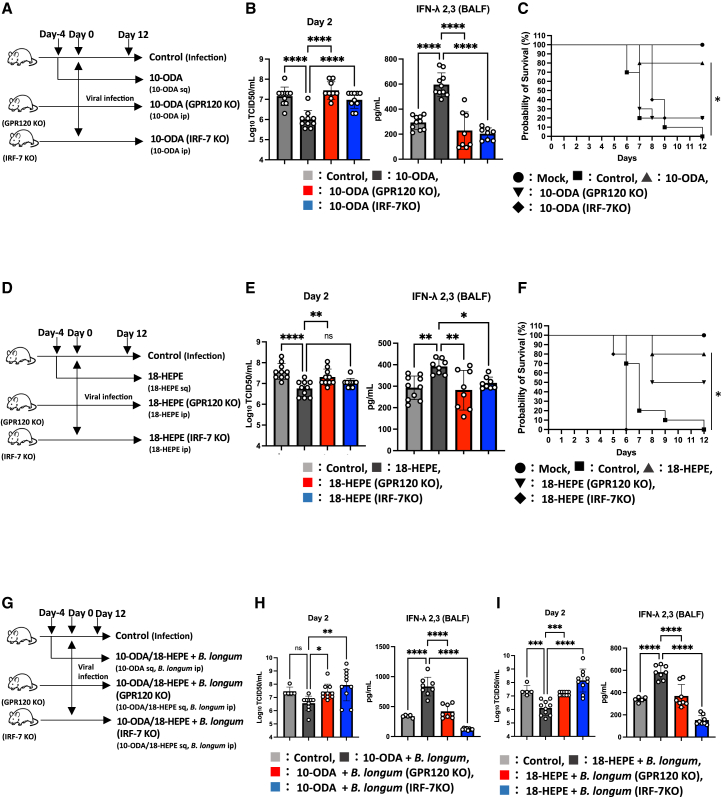
*B. longum* synergistically enhances long-chain fatty acid-induced anti-influenza virus effects via GPR120 (A) The mice received PBS or 10-ODA for 16 days. All groups were infected with the influenza A virus H3N2. Mock, *n* = 10; control, *n* = 10; CBM 588, *n* = 10; 10-ODA (GPR120 KO), *n* = 10; 10-ODA (IRF-7KO); *n* = 10. (B) Viral titer in the lungs on day 2 after influenza virus infection and INF-λ2,3 concentration in bronchoalveolar lavage (BALF). (C) Survival rates. (D) The mice were administered PBS or 18-HEPE for 16 days. All groups were infected with the influenza A virus H3N2. Mock, *n* = 10; control, *n* = 10; CBM 588, *n* = 10; 18-HEPE (GPR120 KO), *n* = 10; 18-HEPE (IRF-7KO); *n* = 10. (E) Viral titer in the lungs on day 2 after influenza virus infection and INF-λ2,3 concentration in BALF. (F) Survival rates. (G) The mice were administered PBS or 10-ODA/18-HEPE + *B. longum* for 16 days. All groups were infected with the influenza A virus H3N2.Control, *n* = 10; 10-ODA/18-HEPE + *B. longum*, *n* = 10; 10-ODA/18-HEPE + *B. longum* (GPR120 KO), *n* = 10; 10-ODA/18-HEPE + *B. longum* (IRF-7KO). (H) Viral titer in the lungs on day 2 after influenza virus infection and INF-λ2,3 concentration in BALF after 10-ODA + *B. longum* treatments. (I) Viral titer in the lungs on day 2 after influenza virus infection and INF-λ2,3 concentration in BALF after 18-HEPE + *B. longum* treatments. The results are presented as mean ± standard deviation (SD). Each dot represents an individual mouse. Results were considered statistically significant when differences were *p* < 0.05 (∗∗∗∗: *p* ≤ 0.0001, ∗∗∗: *p* ≤ 0.001, ∗∗: *p* ≤ 0.01, ∗: *p* ≤ 0.05; ns indicates not significant). See also Figure S6.

Intraperitonially dose of (±)-18-hydroxy-5Z,8Z,11Z,14Z,16E-eicosapentaenoic acid (18-HEPE) results in <30 pg/mL of blood concentration of 18-HEPE 3 h after the dose and showed anti-inflammatory effects.^34^ CBM 588-induced LCFA, 18-HEPE, promotes INF-λ production via IRF-7 activation.^24^ However, whether other LCFAs have similar effects remains unclear. Hence, based on the results of our previous study^24^ and the *in vivo* findings regarding lipid metabolome analyses, we investigated the effects of several other LCFAs on INF-λ production in human epithelial cells (see Figure S6A). Similar to 18-HEPE, 10-oxo-octadecanoic acid (10-ODA) promoted IFN-λ production even under influenza virus infection in a dose-dependent manner (see Figures S6B–S6D). Deletions of *Gpr120* and *Irf-7* genes resulted in IFN-λ down-regulation, similar to the effects of 18-HEPE (see Figures S6E–S6G). In our *in vivo* studies, we observed significantly higher INF-λ2,3 concentration in the BALF and lower virus titers in the lungs in the 10-ODA administration group compared to the control group 2 days post-infection (Figures 6A and 6B). Then, the effect showed a dose-dependent manner (see Figure S6H). In addition, the survival rate in the 10-ODA administration group was significantly higher compared to that of the control group (Figure 6C). Consistent results were observed in the 18-HEPE administration group (Figures 6D–6F). However, 2 days post-infection, the GPR120 KO mice that received 10-ODA or 18-HEPE administrations showed significant IFN-λ2,3 down-regulation in BALF and significantly higher virus titer in the lungs, compared with wild-type mice as well as IRF-7 KO mice, even though both mouse groups received similar 10-ODA or 18-HEPE treatments. Furthermore, to know the additive effects of LCFAs and *B. longum* and to show the importance of GPR120 and IRF-7 for CBM 588-induced anti-virus effects, we evaluated the combination regimens. Consequently, additive anti-influenza virus effects were observed in mice that were co-administered 10-ODA or 18-HEPE with *B. longum* compared to mice that received 10-ODA, 18-HEPE, or *B. longum* separately. However, these additive anti-influenza virus effects were significantly attenuated in GPR120 KO and IRF-7 KO mice (Figures 6G–6I).

## Discussion

Microbiome alterations are known to affect the severity of viral infections^21^^,^^25^^,^^35^^,^^36^^,^^37^^,^^38^^,^^39^^,^^40^; however, the precise mechanisms remain unclear. A higher relative abundance of butyrate-producing bacteria in the gut microbiome is crucial for protection against severe infectious diseases, including respiratory viral infections, compared to gut microbiome diversity.^22^^,^^23^ In this study, we found that the lung microbiome is also important for the anti-influenza virus effects of orally administered butyrate-producing CBM 588.

Gut microbiome-produced butyrate attenuates tissue damage caused by the recruitment of immune cells, such as specific T cells, to the airways during influenza virus infection in mice, and enhances anti-influenza virus effects.^15^^,^^41^^,^^42^ *C. butyricum* is increased in the murine gut with the oral administration of CBM 588, whereas butyrate in the gut is not increased with the oral administration of CBM 588.^43^ However, CBM 588 administration protected the lung epithelial cells and altered LCFA metabolism in the cell. CBM 588-induced LCFA can promote anti-influenza virus effects via IFN-λ upregulation in the lungs.^22^ Hence, we expected that non-butyrate dependent factors, such as LCFAs, could contribute to the IFN-λ upregulation with the lung microbiome in the CBM 588 received group.

The orally administered CBM 588 altered the lung microbiome and induced *Bifidobacterium* species proliferations under influenza virus infection, whereas the time point in our *in vivo* study can still be too an early phase to alter the murine gut microbiome, since mice were sacrificed on 2 days after infection in this study. *Bifidobacterium* species exert anti-influenza virus effects when used as probiotics to activate transient pro-inflammatory host responses.^44^^,^^45^

Patients with influenza virus infection have an altered lung microbiome, compared to healthy volunteers.^26^^,^^46^ However, few study revealed the role of pulmonary bacteria in lung defense. Additionally, as far as our knowledge goes, the relationship between the relative abundance of Bifidobacterium species in the lung microbiome and some factors in the host, such as age, remains unclear. Hence, we are thinking that our data can be one of the pieces of evidence to understand the point.

Furthermore, our study revealed that GPR120 plays an important role in showing anti-influenza virus effects. GPR120, one of the main receptors for LCFAs,^32^ alleviates inflammation and remodeling of airway epithelium partly through Toll-like receptors and TNF-α signaling with p38 to MAPK pathway activation.^47^^,^^48^ Hence, our observations suggest that one of the protective mechanisms of CBM 588 against respiratory viral infections was strongly related to LCFA metabolites.

In particular, IFN-λ regulates mucosal responses to viral infection,^49^^,^^50^ and enhances TJs expression and mucin production to protect the lungs.^51^ Hence, we hypothesize that GPR120, especially in the lung epithelial cells, plays an important role in demonstrating CBM 588-induced anti-influenza virus effects through IFN-λ upregulation.

As oral CBM 588 administration increases *Bifidobacterium* species in the dysbiosis gut,^43^^,^^52^ CBM 588 enhanced the proliferation of *Bifidobacterium* species in lung tissue, and they showed moderate ability to enhance GPR120 expression levels. Hence, we expected that orally administered CBM 588 would promote the proliferation of specific *Bifidobacterium* species in dysbiosis caused by the virus, and that these events could affect CBM 588-induced anti-influenza virus effects through IFN-λ upregulation.

IRFs play an important role in IFN-λ production in the airway epithelium.^53^ Among these, IRF-7 is one of the major regulators of IFN-dependent immune responses^54^^,^^55^^,^^56^ and enhances IFN-λ2,3 productions in mice administered CBM 588 orally.^24^ Additionally, GPR120 KO mice showed the downregulation of IRF-7, as demonstrated in this study. GPCRs share a common structural motif, including the seven transmembrane helices,^46^ and IRF-7 functions in the cytoplasm.^57^ Hence, GPR120 can play an important role in CBM 588-induced anti-influenza virus effects through IRF-7 activation.

Notably, in this study, the co-administration of 10-ODA and *B. longum* showed an additive anti-influenza virus effect as 18-HEPE.^24^ Hence, *B. longum* in the lung microbiome can enhance the susceptibility to LCFAs through the GPR120 upregulation, and that this plays an important role in demonstrating specific LCFA induced anti-influenza virus effects through the IFN-λ upregulation.

### Limitations of the study

Our study revealed important mechanistic insights into how CBM 588 modulates epithelial protective effects against influenza viral pneumonia. Nevertheless, this study has some limitations. First, the murine and human lung microbiomes are not identical.^58^ Further research is required to investigate whether these results can be reproduced in humans. Additionally, as far as our knowledge, few study evaluated the feasibility of nasal *B. longum* administration in humans and any safety/efficacy. Hence, further study to evaluate them are also needed. Secondly, we did not identify the precise mechanisms by which orally administered CBM 588 enhanced the proliferation of specific *Bifidobacterium* species. However, few previous studies have shown that CBM 588 can enhance *Bifidobacterium* species proliferations,^43^^,^^52^ and we need to further explore the mechanisms in more detail. CBM 588 produced protein may be a candidate to affect the proliferations of bacteria, because proteinase K can affect the effect. However, we have not reached a conclusion. Hence, our project is going to investigate the point with LC-MS/MS, and so forth, to reveal the mechanism to reshape the lung microbiome with orally administered CBM 588. Finally, in our microbiome study, the mock (+ CBM 588) group data are lacking. Hence, we do not know whether oral CBM 588 administration would alter lung microbiome under non-virus infection or not.

In conclusion, we revealed that the importance of the lung microbiota in early immune defense against influenza virus infection, and a particular lung microbiome, is important for butyrate-producing CBM 588-induced anti-influenza viral effects. CBM 588 altered the lung microbiome, and these alterations affected GPR120 expression levels through the *Bifidobacterium* species proliferations. In parallel, orally administered CBM 588 upregulates certain LCFAs that could promote IFN-λ production, such as 10-ODA and 18-HEPE. These increased the susceptibility of lung epithelial cells to LCFAs and resulted in the enhanced anti-influenza virus effects through the IFN-λ upregulation.

Moreover, we revealed the mechanism of orally administered CBM 588-induced anti-influenza virus effects and provided insights into targets for respiratory viral infection treatment and prophylaxis. We are currently investigating the antiviral effects against other respiratory viruses, such as severe acute respiratory syndrome coronavirus 2 (SARS-CoV-2). Additionally, this study suggests the potential for non-invasive and inexpensive methods to protect against other respiratory virus infections, in addition to influenza viruses.

## Resource availability

### Lead contact

Further information and requests for resources and reagents should be directed to and will be fulfilled by the lead contact, Hiroshige Mikamo (mikamo@aichi-med-u.ac.jp).

### Materials availability

The bacterial strains used in this study are available from the lead contact with a completed materials transfer agreement.

### Data and code availability

• LC-MS/MS data have been deposited at MetaboLights MTBLS4590 and are publicly available (https://www.ebi.ac.uk/metabolights/reviewer18cf2487-8bc9-4b84-b575-314f8fc149d5).
• 16S rRNA amplicon sequence data have been deposited in the DNA DataBank of Japan under BioProject PRJDB13288, PRJDB13290, PRJDB13291, PRJDB13319, and PRJDB13320, respectively.
• This paper does not report original code.
• Any additional information required to reanalyze the data reported in this paper is available from the lead contact upon request.

## Acknowledgments

MIYARISAN Pharmaceutical Co., Ltd. provided CBM 588 powder. The Division of Laboratory Animal Research at Aichi Medical University provided facilities and supported the animal experiments. The Division of Advanced Research Promotion at Aichi Medical University provided technical instructions and assistance with this study. This work was supported by 10.13039/501100001691JSPS KAKENHI Grant Numbers 22K08591, 23K07954, and 25K11773, Aichi Medical University Research Unit grant aid, 10.13039/100007449Takeda Science Foundation, and the Institute for Fermentation, Osaka IFO (L-2024-3-002). Finally, we thank Dr. Yoshihiro Kawaoka (University. Tokyo, Japan) to provide the viruses and MDCK cells.

## Author contributions

Conceptualization, M.H.; methodology, M.H., M.Y., T.A., and A.O.; investigation, M.H. (all experiments), C.Y., S.H., and A.M. (r16S sequencing), N.A., T.A., H.K., Y.S., and T.U. (mass-spectrum assays), A.M., and K.I. (flow cytometry), H.K., J.H., and T.M. (tissue evaluation); formal analysis, S.H. and M.H.; writing – original draft, M.H.; writing – review and editing, M.Y., M.H.; funding acquisition, M.H., K.O., M.T., and H.M.; resources, H.M.; and supervision, M.H and M.Y.

## Declaration of interests

M.T., K.O., T.A., C.Y., S.E., A.M., and S.H. are employees of MIYARISAN Pharmaceutical Co., Ltd. H.M. received research funding from Miyarisan Pharmaceutical Co., Ltd.; Honorarium/Consulting Fee from Miyarisan Pharmaceutical Co., Ltd. The other authors declare no competing interests.

## STAR★Methods

### Key resources table

**Table:** 

REAGENT or RESOURCE	SOURCE	IDENTIFIER
**Antibodies**		

IL-28A/IFN-lambda 2 anti-Mouse Monoclonal Ab (625616)	RSD	MAB4635; RRID: AB_10718823
IL-28B/IFN-lambda 3 anti-mouse Monoclonal Ab (244710)	RSD	MAB1789; RRID: AB_2233689
FITC-labeled anti-CD4 Ab (RM4-5)	BD Biosciences	561835; RRID: AB_394582
Per-CP-labeled anti-CD3 Ab (145-2C11)	BD Biosciences	561089; RRID: AB_394599
PE-labeled anti-CD8 Ab (53-6.7)	BD Biosciences	561095; RRID: AB_394571
Rat IgG2b Isotype Control antibody (LTF-2)	Thermo Fisher Scientific	02-9288; RRID: AB_2532966_
APC-labeled anti-CD326 (Ep-CAM) Ab (G8.8)	BioLegend	118213; RRID: AB_1134105
PB-labeled anti-CD45 Ab (30-F11)	BioLegend	103128; RRID: AB_493715
FITC-labeled anti-CD11c Ab (N418)	BioLegend	117305; RRID: AB_313774
PerCP anti-I-A/I-E (MHC II) Ab (M5/114.15.2)	BioLegend	107623; RRID: AB_893586
BB700-labeled anti-NK1.1 Ab (PK136)	BD Horizon	566503; RRID: AB_2744491
BV421-labeled anti-CD49b Ab (DX5)	BD Horizon	563063; RRID: AB_2737983
BV421-labeled anti-CD11 Ab (M1/70)	BioLegend	117330; RRID: AB_11152949
FITC-labeled anti-neutrophil Ab (7/4)	abcam	ab53453; RRID: AB_881408
eFluour-labeled anti-Ly-6G Ab (1A8-Ly6g)	invitrogen	48-5931-82; RRID: AB_2637124
FITC-labeled anti-F4/80 Ab	Abcam;	ab60343; RRID: AB_941505
Anti-GPR120, Rabbit-Poly	GENETEX	GTX100364; RRID: AB_1240849

**Bacterial and virus strains**		

Bacterium: *Clostridium butyricum* MIYAIRI 588	MIYARISAN Pharmaceutical Co.	N/A
Virus: Influenza virus (A/Aichi/2/68) (H3N2)	Yoshihiro Kawaoka	N/A
*Anaerotruncus colihominis*	JCM	15631
*Bifidobacterium infantis* AMU-1	This paper	N/A
*Bifidobacterium longum* AMU-1	This paper	N/A
*Bifidobacterium adolescents* AMU-1	This paper	N/A
*Bifidobacterium bifidum* AMU-1	This paper	N/A
*Bifidobacterium breve* AMU-1	This paper	N/A
*Bifidobacterium catenulatum* AMU-1	This paper	N/A
*Bifidobacterium thermophilis* AMU-1	This paper	N/A
*Akkermansia muciniphila*	JCM	30893
*Bilophila wadsworthia*	JCM	35487
*Cereibacter azotoformans*	JCM	9340
*Muribaculum intestinale*	JCM	33112
*Parasutterella excrementihominis*	JCM	15078
*Parasutterella secunda*	JCM	16078
*Lactococcus lactis* AMU-1	This paper	N/A
*Lactobacillus iners* 22-2590	This paper	N/A
*Lactobacillus crispatus* 8-1	This paper	N/A
*Limosilactobacillus rauteri 1506*	This paper	N/A
*Lactobacillus gasseri* AMU-1	This paper	N/A
*Limosilactobacillus fermentum YB-1839*	This paper	N/A
*Peptostreptococcus anaerobius* 19	This paper	N/A
*Streptococcus pneumoniae* KY-9	This paper	N/A
*Staphylococcus aureus*	ATCC	25923
*Enterobacter cloacae* 20-5694	This paper	N/A
*Escherichia coli*	ATCC	25922
*Haemophilus influenzae AMU-3086*	This paper	N/A
*Klebsiella pneumoniae*	ATCC	700603
*Prevotella intermedia* 335	This paper	N/A
*Pseudomonas aeruginosa* AMU 13-4632	This paper	N/A
*Agathobacter rectalis*	JCM	17463
*Alistipes putredinis*	JCM	16772
*Anaerofustis stercorihominis*	ATCC	BAA-858
*Anaerococcus vaginalis*	JCM	8138
*Anaerostipes caccae*	JCM	13470
*Anaerostipes caccae*	JCM	35490
*Anaerotruncus colihominis*	JCM	15631
*Anaerotruncus colihominis*	JCM	31255
*Clostridium butyricum*	ATCC	19398
*Clostridium perforingens*	JCM	3817
*Clostridium perforinge*	JCM	1290T
*Clostridium symbiosum*	JCM	1297
*Coprococcus comes*	JCM	31264
*Eubacterium desmolans*	ATCC	43058
*Eubacterium hallii*	ATCC	27751
*Eubacterium limosum*	JCM	6421
*Eubacterium limosum*	JCM	6501
*Eubacterium limosum*	JCM	9978
*Eubacterium limosum*	JCM	10283
*Fusobacterium animalis*	JCM	11025
*Faecalibacterium prausnitzii*	JCM	39207
*Faecalibacterium prausnitzii*	JCM	39209
*Fusobacterium nucleatum*	JCM	6328
*Fusobacterium nucleatum*	JCM	8532
*Fusobacterium polymorphum*	JCM	12990
*Fusobacterium vincentii*	JCM	11023
*Holdemanella biformis*	JCM	10412
*Propionibacterium acidifaciens*	JCM	16571
*Pseudoramibacter alactolyticus*	JCM	6480
*Roseburia intestinalis*	JCM	31262
*Roseburia intestinals*	JCM	17583
*Roseburia inulinivorans*	JCM	17584
*Roseburia inulinivorans*	JCM	31260
*Clostridium symbiosum* AMU 22-3629	This paper	N/A
*Megasphaera micronuciformis* AMU 21-1966	This paper	N/A
*Clostridium butyricum* MI-56	This paper	N/A
*Clostridium butyricum* MI-64	This paper	N/A
*Clostridium butyricum* MI-65	This paper	N/A
*Clostridium butyricum* MI-20	This paper	N/A
*Eubacterium limosum* AMU 22-2631	This paper	N/A
*Eubacterium limosum* AMU 22-3666	This paper	N/A
*Eubacterium limosum* AMU 22-1127	This paper	N/A
*Eubacterium limosum* AMU 22-666	This paper	N/A
*Eubacterium limosum* AMU 21-2506	This paper	N/A
*Eubacterium limosum* AMU 21-4266	This paper	N/A
*Clostridium perforingens 21004190*	This paper	N/A
*Clostridium perforingens* S13	This paper	N/A
*Clostridium perforingens* 21004457	This paper	N/A
*Clostridium perforingens* 21004283	This paper	N/A
*Clostridium perforingens* 21004279	This paper	N/A
*Anaerococcus vaginalis* 24-781	This paper	N/A
*Anaerococcus vaginalis* 23-3064	This paper	N/A
*Anaerococcus vaginalis* 23-3301	This paper	N/A
*Anaerococcus vaginalis* 23-3265	This paper	N/A
*Anaerococcus vaginalis* 23-3843	This paper	N/A
*Fusobacterium varium* 21-4275	This paper	N/A
*Fusobacterium varium* 22-218	This paper	N/A
*Fusobacterium varium* 22-220	This paper	N/A
Fusobacterium varium 22-1632	This paper	N/A
*Fusobacterium varium* 22-118	This paper	N/A
*Fusobacterium nucleatum* 24-780	This paper	N/A
*Fusobacterium nucleatum* 24-911	This paper	N/A
*Fusobacterium nucleatum* 24-883	This paper	N/A
*Fusobacterium nucleatum* 24-910	This paper	N/A
*Clostridium tertium*	JCM	6289
*Clostridium beijerinckii*	ATCC	858
*Clostridium beijerinckii*	ATCC	6014
*Clostridium beijerinckii*	ATCC	25752
*Clostridium beijerinckii*	ATCC	6015

**Chemicals, peptides, and recombinant proteins**		

Penicillin-Streptomycin-Amphotericin B Suspension (100×)	Wako	161-23181
MEM (10×)	gibco	11430-030
MEM Amino Acids (50×)	gibco	11130-051
MEM Vitamin Solution (100×)	gibco	11120-052
Distilled water	gibco	15230204
Newborn Calf Serum, heat inactivated	gibco	26010066
0.25% Trypsin (1×)	gibco	15050065
Sodium bicarbonate solution (7.5% NaHCO3)	Sigma	S8761-100mL
Bovine Serum Albumin Solution (7.5%)	Sigma	A8412-100ML
L-Glutamine Solution (50x)	Sigma	G7513-100ML
0.5%-Trypan Blue Stain Solution	Nacalai tesque	29853-34
Crystal Violet	Nacalai tesque	09803-62
F-12K Medium	ATCC	ATCC 30-2004
Collagenase, type IV	Worthington Collagenase	LS004209
Recombinant DNase I (RNase-free)	Takara	2270A
RPMI 1640	Thermo Fisher Scientific	11875-085
HBSS	Thermo Fisher Scientific	14025-76
EDTA	Sigma	E5134-50g
Percoll	GE Healthcare	17-0891-02
Formalin solution neutral buffered, 10%	SIGMA	HT501128-4L
USDA-Grade Certified Fatal Bovine Serum [FBS]	Cosmo bio	04-001-1A
Isoflurane	Pfizer	114-133403
Kanamycin sulfate	Sigma-Aldrich	K1377
Clindamycin	Pfizer	876112
Vancomycin	Pfizer	N/A
Metronidazole	Pfizer	876419
(±)18-HEPE	CAY	32840
10-oxo-octadecanoic acid	AstaTech Inc.	A11138
Myristic acid	Sigma	M312
Linoleic acid	Sigma	L1376
Oleic acid	Sigma	O1008
TUG-891	CAY	17035
Eicosa pentaenoic acid	CAY	99110
Stearic acid	TCI	S0163-25G
Palmitoreic acid	CAY	10009871
Prostaglandin E1	Avanti	745-65-3
Prostaglandin E2	CAY	14010
Prostaglandin F1α	Avanti	745-62-0
10-hydroxy-octadecanoic acid	Ambeed Inc.	AMBH93D5360
TUG-891	CAY	17035
FITC-dextran	Sigma	FD4-1G

**Critical commercial assays**		

NucleoSpin® RNA (Total RNA isolation Kit)	MACHEREY-NAGEL	740955.50
Fixation/Permeablization Kit	BD Biosciences	554714
High-Capacity RNA-to-cDNA Kit	Thermo Fisher Scientific	4387406
SYBAR Green PCR Master Mix	Thermo Fisher Scientific	A25742
VeriKine™ Mouse IFN-α detection ELISA Kit	PBL	42120-1
Mouse IFN-β Quantikine ELISA Kit	RSD	MIFNB0
LEGEND MAX™ Mouse IFN-γ ELISA Kit	Biolegend	430807
Mouse IL-28 ELISA Kit	abcam	ab100708
LEGEND MAX™ Mouse IL-6 ELISA Kit	Biolegend	431307
LEGEND MAX™ Mouse TNF-α ELISA Kit	Biolegend	430907
Human IL-29 (IFN-λ1) ELISA Kit	Biolegend	446307
Human IL-28A (IFN-λ2) ELISA Kit	RayBiotech	ELH-IL28A-1
Human IL-28B/IFN-lambda 3 Quantikine ELISA Kit	R&D Systems	D28B00
Human Free Fatty Acid Receptor 4 (FFAR4) ELISA Kit	CLOUD-CLONE	SEG697HU

**Deposited data**		

LC-MS/MS data	MetaboLights	MTBLS4590 (https://www.ebi.ac.uk/metabolights/reviewer18cf2487-8bc9-4b84-b575-314f8fc149d5)
Miseq data	DNA Data Bank of Japan under BioProject	PRJDB13288, PRJDB13290, PRJDB13291, PRJDB13319, PRJDB13320

**Experimental models: Cell lines**		

Human wild-type A549 cell line	abcam	ab255450
Human GPR120 knockout A549 cell line	This paper	N/A
Human IRF-7 knockout A549 cell line	abcam	ab267210
MDCK cell line	Yoshihiro Kawaoka	N/A

**Experimental models: Organisms/strains**		

Mouse: BALB/c	Charles River Laboratories Japan, Inc.	N/A
Mouse: C57BL/6J	Charles River Laboratories Japan, Inc.	N/A
Mouse: *gpr120*^-/-^ mice	The RIKEN BioResource Research Center	RBRC10195
Mouse: *Irf7*^-/-^ mice	The RIKEN BioResource Research Center	RBRC01420

**Oligonucleotides**		

See Table S1 for primers	N/A	N/A

**Software and algorithms**		

Prism v9.0	GraphPad Software	https://www.graphpad.com/scientific-software/prism/
FlowJo v10.07	TreeStar Inc	https://www.flowjo.com/
Xcalibur 4.1	Thermo Fisher Scientific	https://www.thermofisher.com/jp/ja/home/industrial/mass-spectrometry/liquid-chromatography-mass-spectrometry-lc-ms/lc-ms-software/lc-ms-data-acquisition-software/xcalibur-data-acquisition-interpretation-software.html
Compound Discoverer3.1.1.12	Thermo Fisher Scientific	https://www.thermofisher.com/jp/ja/home/industrial/mass-spectrometry/liquid-chromatography-mass-spectrometry-lc-ms/lc-ms-software/multi-omics-data-analysis/compound-discoverer-software.html

**Other**		

TissueLyser II	QIAGEN	85300
Stainless beads, 3mm	TAITEC	0068218-000
100-μm cell strainer	PLS	43-50100-03
FACSCant™ II	BD Biosciences	N/A
Step One Plus (Real time PCR system)	Thermo Fisher Scientific	N/A
Liquid chromatography-tandem mass spectrometry (LC-MS/MS)	Thermo Fisher Scientific	N/A
FACSCant™ II	BD Biosciences	N/A
FACSAria III	BD Biosciences	N/A
Freeze dryer	EYELA	FDU-2200
Centrifugal evaporator	EYELA	CVE-3110
Cooling trap device	EYELA	UT-2000
Diaphragm vacuum pomp	EYELA	NVP-2100
Vacuum pomp	EYELA	GCD-136XN
Ball mill	Retsxh	MM 400
Zirconia ball	NIKKATO CORPORATION	YTZ-0.3
Centrifuge	HITACHI	CF16RN
Vortex mixer	Scientific Industries	SI-0286
Methanol	FUJIFILM Wako Pure Corporation	134-14523
Acetonitrile	FUJIFILM Wako Pure Corporation	018-19853
BD Microtainer SST Tubes	BD Biosciences	365967
SpectraMax iD5®	Molecular Devices	N/A

### Experimental model and study participant details

#### Mice

Eight- to nine-week-old specific pathogen-free (SPF) female BALB/c (wild type and *Gpr120*^-/-^ mice) and C57BL/6J (wild type and *Irf-7*^-/-^ mice) mice were purchased and housed in the Animal Facility at Aichi Medical University. This study used only female mice. Hence, we could not report the influences of sex on our study data. The *in vivo* studies were approved by the Ethics Committee of the Aichi Medical University (#2024-60) and were performed according to ARRIVE guidelines 2.0 (https://arriveguidelines.org). Additionally, we followed the American Veterinary Medical Association (AVMA), Guidelines for the Euthanasia of Animals (2020) (https://www.avma.org) and the Japanese Association for Laboratory Animal Medicine’s Publication Guide for the Use of Laboratory Animals and Care for Animal Experimental Procedures (https://www.jalam.jp/).

#### Cell line

Human wild-type A549 cell line (abcam, ab255450), and Human IRF-7 knockout A549 cell line (abcam, ab267210) were purchased. Human GPR120 knockout A549 cell line was made for this study with CRISPR/Cas9 described as below, and MDCK cell line were kindly provided from Yoshihiro Kawaoka (University. Tokyo, Japan).

### Method details

#### Murine influenza virus infection model

Influenza virus strain (A/Aichi/2/68 (H3N2)) was kindly provided by Yoshihiro Kawaoka (University of Tokyo, Japan). All viral infection experiments were conducted as previously described.^24^ Frozen aliquots of influenza viral stock (1.1×10^7^ pfu/mL) were thawed and diluted to 1.1×10^4^ pfu/mL with PBS. Then, 50 μL of virus solution was administered intranasally to each mouse under anesthesia with isoflurane inhalation solution (Pfizer Japan Inc.). As previously described, viral infectivity in the lungs was assessed by titration of 50% tissue culture infectious dose (TCID_50_) in Madin–Darby canine kidney cells (MDCK).^24^ During the study period, the mice’s body weight was recorded daily as the change (%) from the initial body weight (day 0).

#### Morphologic and immunohistochemical analysis of the lungs

At the sampling points, mice from each group were sacrificed to identify histological changes in the lung tissue. The mice were euthanized by an overdose of CO_2_ followed by cervical dislocation, and the organs were harvested. Fixed sections of the lung tissues were embedded in paraffin. These tissues were then cut into 3-μm sections, and stained with hematoxylin and eosin (H&E) for histological analysis via light microscopy. Immunohistochemistry was performed using unstained sections obtained from formalin-fixed, paraffin-embedded blocks, deparaffinized in xylene, and rehydrated in ethanol. Antigen retrieval was conducted by autoclaving at 120 °C for 10 min, followed by blocking endogenous peroxidase with 0.3% hydrogen peroxide. Then, these samples were incubated with an unconjugated rabbit anti-MUC5AC polyclonal antibody (bs-7166R, 1:100) overnight at 4 °C, followed by a conjugated secondary and 3,3'-diaminobenzidine (DAB) staining.

#### Pathologic assessments

Lung tissues were evaluated for histological changes as previously described.^24^ We assessed the tissues for: (1) mucosal edema and hemorrhagic congestion, (2) damage to epithelial cells, and (3) tissue infiltration with neutrophil margination. A skilled pathologist blinded to the experimental condition evaluated the samples. To compare the severity of pneumonia, a score (0–3) was assigned to each parameter. Subsequently, the combined scores were evaluated. To determine *in vivo* intestinal permeability, fluorescein isothiocyanate (FITC)-dextran (Sigma-Aldrich, Tokyo, Japan) was administered intranasally to mice (0.44 mg/100 g body weight). After 1 h, mice were euthanized by an overdose of CO_2_ followed by cervical dislocation, and blood was collected via cardiac puncture. Serum, separated from whole blood using BD Microtainer SST Tubes (BD Biosciences, Franklin Lakes, NJ, USA), was diluted by an equal volume of PBS (pH 7.4), and 100 μL of this diluted serum was dispensed into a 96-well microplate. The concentration of FITC in the serum was determined by fluorescent spectrophotometry at an excitation wavelength of 485 nm and an emission wavelength of 528 nm using serially diluted FITC-dextran as a standard.

#### Bacterial treatments

Miyarisan Pharmaceutical Co. Ltd. (Tokyo, Japan) kindly provided the bacterial powder form of *Clostridium butyricum* MIYAIRI 588 (CBM 588) (2.2×10^10^ CFU/g: Lot 61GT). As in our previous study,^24^ mice received a daily oral dose of CBM 588 at 500 mg/kg/day (3.4×10^8^ CFU/mice/day) via a sonde from day -4 to day 12. Additionally, mice received nasal administrations of suspensions (50 μL) containing each *Bifidobacterium* species at 1.0-3.0×10^8^ CFU/mL/mouse/72 h. To administer the antibiotic mixture, clindamycin (Pfizer Japan Inc.), vancomycin injection (Pfizer Japan Inc.), metronidazole (Pfizer Japan Inc.), and analytical-grade kanamycin sulfate (K1377, Sigma-Aldrich) were purchased and diluted in PBS to a stock concentration of 500 mg/mL. Prior to each *in vivo* experiment, CBM 588 powder was weighed and reconstituted with sterile water. The stock solution of the Abx was diluted with PBS to achieve the desired concentration, and 50 or 100 μL of the solution was administered intranasally or orally to each mouse under anesthesia with isoflurane inhalation solution (Pfizer Japan Inc.).

#### Treatment with chemicals and antibodies

Analytical grade 10-ODA was purchased from AstaTech Inc. (Bristol, PA, USA), dissolved in dimethyl sulfoxide (DMSO), and diluted with PBS (pH 7.4) to 25 μg/mL prior to each experiment. For 18-HEPE administration, analytical grade 18-HEPE was purchased from CAY (Ann Arbor, MI, USA). diluted with PBS (pH 7.4) to 25 μg/mL immediately before use, and administered intraperitoneally (ip) at 5 μg/mice once on days -3, 0, 3, 6, and 9.^62^

#### Microbiome analysis

Microbiome analysis was conducted as described previously.^43^^,^^52^^,^^63^^,^^64^^,^^65^ Briefly, BALF and fecal samples were used to determine the lung and gut microbiome compositions. MiSeq sequencer (Illumina) was used for 16S rRNA sequencing (V3-V4 regions) and the data were analyzed using the Quantitative Insights into Microbial Ecology (QIIME 2) pipeline.^66^ Chimeric sequences were removed using the USEARCH software.^67^ Based on 97% sequence similarity at the species level, sequences were clustered into operational taxonomic units (OTUs) using UCLUST.^66^ Representative sequences of each out were aligned using PyNAST.^66^ α- and β-diversity was measured using QIIME 2 default scripts.^68^ Each colored point represents a sample from a single mouse, with colors indicating different treatments.

#### Isolations of lymphocytes from lung tissue and lung epithelial cells

Lymphocytes were isolated from the lung tissue according to our previous methods.^24^^,^^69^ Briefly, after perfusion with PBS, the lungs were harvested and cut into small pieces. Subsequently, they were incubated in Hank’s balanced salt solution (HBSS) (containing ethylenediaminetetraacetic acid disodium salt, 2-hydrate (EDTA); 5 mM), and fetal bovine serum (FBS); 1.5%) for 20 min at 37 °C with constant agitation. After removing fat tissue and epithelial cells, the tissues were digested with 1 mg/mL collagenase type IV (Worthington Biochemical Corporation, Lakewood, NJ, USA) and resuspended in 40% Percoll (GE Healthcare, Chicago, IL, USA) before being overlaid on 80% Percoll. Interface cells were collected after centrifugation (800 × g for 20 min at 20 °C). These cells were used as lymphocytes. Lung epithelial cells were isolated from the lung tissue according to previously described methods.^24^^,^^69^ Isolated lungs were cut into small pieces and incubated in HBSS (containing EDTA: 5 mM) and FBS: 1.5%) for 20 min at 37 °C with constant agitation to disrupt the epithelial integrity. The liberated cells were resuspended in 20% Percoll™, followed by an overlay with 40% Percoll™ in a tube. Interface cells were collected after centrifugation (780 × g for 20 min at 25 °C), and the cells were sorted using FACSAria (BD Biosciences). Then, the following antibodies were used: APC-labeled anti-CD326 (Epi-CAM) Ab (118213, BioLegend); Pacific Blue labeled anti-CD45 Ab (103125, BioLegend); and 1 μg/mL ionomycin (Sigma-Aldrich).^24^ After that, cells (CD326^+^/CD45^-^) were used as lung epithelial cells.

#### Flow cytometry analysis

To determine the abundance of T cells, monocytes, neutrophils, macrophages, natural killer cells, and macrophages in the lymphocytes derived from the lungs and lung epithelial cells, we used flow cytometry (FACSCantTM II, BD Biosciences). The following antibodies were also used: FITC-labeled anti-CD4 Ab (561835, BD Biosciences); Per-CP-labeled anti-CD3 Ab (561089, BD Biosciences); PE-labeled anti-CD8 Ab (561095, BD Biosciences); BB700-labeled anti-NK1.1 Ab (566503, BD Horizon); BV421-labeled anti-CD49b (DX5) Ab (563063, BD Horizon); BV421-labeled anti-CD11b Ab (117330, BioLegend); FITC-labeled anti-neutrophil [7/4] Ab (ab53453, abcam); eFluour-labeled anti-Ly-6G Ab (48-5931-82, invitrogen); FITC-labeled anti-F4/80 Ab (ab60343, abcam); APC-labeled anti-CD326 Ab (118213, BioLegend); Pacific Blue labeled anti-CD45 Ab (103125, BioLegend); and 1 μg/mL ionomycin (Sigma-Aldrich).^22^ The data were analyzed using FlowJo software (TreeStar Inc., Williamson Way Ashland, OR, USA).

#### Quantitative reverse transcription polymerase chain reaction

Quantitative ***reverse transcription*** PCR (qRT-PCR) was performed according to our previously reported methods.^24^^,^^52^^,^^63^^,^^66^ Total RNA was extracted from the isolated mouse lung epithelial cells using an RNA isolation kit (MACHEREY-NAGEL). A high-capacity RNA-to-cDNA kit (Thermo Fisher Scientific) and PowerUp™ SYBRTM Green PCR Master Mix (Applied Biosystems) were used for cDNA synthesis and qPCR analysis, respectively. Table S1 list the primer sequences used in this study. ΔΔCt method was used to evaluate target gene expression. Relative RNA expression levels of each target gene were normalized to those of the control group (RQ).

#### SCFA and LCFA metabolomic analysis

SCFA- and LCFA metabolomic analyses were conducted as described previously.^24^^,^^52^^,^^63^^,^^64^^,^^65^^,^^70^ In brief, samples from the mice were cryopreserved (stored at -80 °C) immediately. For SCFAs analysis, SCFAs (acetate, propionate, n-butyrate, iso-valerate, succinate, and lactate) in BALF or feces were measured using high-performance liquid chromatography (Prominence, Shimadzu, Kyoto, Japan) with a post column reaction and detector (CDD-10A, Shimadzu). The system included two tandemly-arranged columns (Shim-pack SCR-102 (H), 300 mm × 8 mm ID; Shimadzu), and a guard column (Shim-pack SCR-102 (H), 50 mm × 6 mm ID; Shimadzu) using a mobile phase (5 mM p-toluene sulfonic acid) and a reaction solution (5 mM p-toluene sulfonic acid, 100 μM EDTA, and 20 mM Bis–Tris). For LCFA analysis, lung epithelial cells were lyophilized, and 10 mg was weighed after ball milling. We used a Vanquish UHPLC system (Thermo Fisher Scientific) coupled with a Q Exactive Focus (Thermo Fisher Scientific), equipped with an electrospray ionization device for liquid chromatography-tandem mass spectrometry (LC-MS/MS) and Orbitrap LC-MS/MS analyses. An Acclaim RSLC120 C18 column (Thermo Fisher Scientific) was used for the analysis. Especially for LCFAs, we conducted comprehensive analysis without standards for each metabolite to reveal their real concentrations as our previous study.^63^^,^^64^ To show the results, metabolites showed significantly differences (*P* < 0.05) on their peak area between control and CBM 588 administration group were picked up, and the data are expressed as fold changes of each peak area.

#### *In vitro* stimulation by LCFAs, and bacterial species

*In vitro* stimulation by LCFAs or bacterial species analysis was conducted as previously described.^24^ After human alveolar epithelial A549 (mycoplasma-free) cells were stimulated with 18-HEPE (0: control, 5, 50, and 500 nM, and 5 μM; CAY), myristic acid (500 nM; Sigma-Aldrich), linoleic acid (500 nM; Sigma-Aldrich), oleic acid (500 nM; Sigma-Aldrich), or TUG-891 (500 nM; CAY); eicosa pentaenoic acid: EPA (500 nM; CAY), stearic acid (500 nM; TCI), palmitoreic acid (500 nM; CAY); prostaglandin E1: PGE1 (500 nM; Avanti); prostaglandin E2: PGE2 (500 nM; CAY); prostaglandin F1α: PGF1α (500 nM; Avanti), 10-hydroxy-octadecanoic acid (500 nM; Ambeed Inc.); 10-ODA (0: control, 5, 50, and 500 nM, and 5 μM; AstaTech Inc.); and TUG-891 (500 nM; CAY) in F-12K medium (ATCC) containing 10% heat-inactivated FBS, streptomycin, penicillin, and amphotericin B (Wako) for 48 h^24^; A549 cells were infected with influenza virus H3N2 (MOI = 0.01) for 1 h and the supernatant was removed and replaced with fresh F-12K media containing fatty acids and 10% heat-inactivated FBS and antimicrobials for 48 h. For bacterial exposures, we selected 30 bacterial species —20 representative bacterial strains based on the lung microbiome data at genus level (Figure 2C) and 10 additional bacterial species frequently detected in human respiratory samples.^26^^,^^28^^,^^71^^,^^72^ A549 cells were infected with influenza virus H3N2 (MOI = 0.01) for 1 h, after which the supernatant was removed and replaced with the adjusted F-12K medium containing each bacterial species (1.0 × 10^9^ CFU/mL) and 10% heat-inactivated FBS and antimicrobials for 48 h. Next, the culture supernatants were harvested and we measured cytokine levels using enzyme-linked immunosorbent assay (ELISA) or specific gene expression levels using RT-PCR. For bacterial incubation medium exposure, we prepared supernatants of each bacterium after 24 h of anaerobic incubation in Gifu anaerobic broth (GAM). A549 cells were infected with influenza virus H3N2 (MOI = 0.01) for 1 h, followed by supernatant removal and replacement with adjusted F-12K medium, Supernatants of butyrate-producing bacteria cultures (0–20% of all medium), 10% heat-inactivated FBS, and antimicrobials were subsequently added. The cells were t incubated anaerobically for 48 h. Total RNA was extracted from the residual A549 cells using an RNA isolation kit (Macherey-Nagel) according to the manufacturer’s protocol.

#### GPR120 knockout using the CRISPR/Cas9 system

The CRISPR/Cas9 system was used to disrupt GPR120 expression in the A549 human lung epithelial cell line. The pSpCas9(BB)-2A-GFP (PX458) vector was gifted by Feng Zhang (Addgene plasmid # 48138). A single guide RNA (sgRNA) sequence was selected using E-CRISP (http://www.e-crisp.org/E-CRISP/index.html). The sgRNA sequence for *FFAR4* (GPR120) Exon 1 was 5′- GAATGTCCCCTGAATGCGCG, which corresponds to the 3′ sequence of the initiation codon of *GPR120*. The plasmid expressing hCas9 and GPR120 sgRNA was prepared by ligating oligonucleotides into the BbsI site of PX458 (*GPR120*/PX458). To generate a *GPR120* knockout clone, 1 μg *GPR120*/PX458 plasmid was nucleofected into A549 cells (1 × 10^6^ cells) using a 4D-Nucleofector instrument (Lonza Japan, Tokyo, Japan). After 3 days, cells expressing green fluorescent protein were sorted using FACSAria (BD Biosciences), and single-cell cloning was performed. A single clone was selected, expanded, and used for biological assays. For sequence analysis of *GPR120* exon 1, the following primer set was used to amplify the genomic DNA: 5′- AGTCGCCTCCCAGATGAGCACT and 5′- CCCTTGACGTCGGAGAAGAAG.

#### Western blot analysis

Cells were lysed in a lysis buffer (0.5% NP-40, 1% TritonX-100, 150 mM NaCl, and 1 mM EDTA in 20 mM Tris, pH 7.5) containing a protease inhibitor cocktail (Nacalai Tesque, Kyoto, Japan) and a PhosSTOP phosphatase inhibitor cocktail (Roche Diagnostics). After incubating for 10 min on ice, the cell lysates were centrifuged at 12,000×*g* for 10 min at 4 °C and the supernatants were recovered. Protein content sample was measured using the Bradford method, and 70 μg aliquots of protein were separated in a Mini-PROTEAN TGX Precast Gel (Bio-Rad Laboratories) and transferred to a nitrocellulose membrane (Bio-Rad Laboratories). The membrane was incubated with the indicated antibodies and horseradish peroxidase (HRP)-labeled secondary antibodies. The signal was then visualized using an enhanced Chemi-Lumi One Super (Nacalai Tesque) and detected using ChemDoc XRS+(Bio-Rad Laboratories). The primary antibodies used were GTX100364 Anti-GPR120, Rabbit-Poly (GENETEX), and anti-GAPDH (Abcam), while the secondary antibodies were goat anti-mouse IgG-HRP or goat anti-rabbit IgG-HRP (Santa Cruz Biotech).

### Quantification and statistical analysis

All statistical analyses were conducted using GraphPad Prism 9 (GraphPad Software, San Diego, CA, USA), as described in our previous study.^3^ The Mann–Whitney U test or Student’s *t*-test was used to evaluate differences between the two groups for non-parametric and parametric data. The Kruskal–Wallis test (non-parametric), followed by Dunn’s test as a post hoc test, or one-way analysis of variance (ANOVA) (parametric), followed by Bonferroni correction, was conducted for multiple group comparisons. Kaplan–Meier curves and the log-rank test were used to perform survival analysis and compare between groups. Results were considered statistically significant when differences were p < 0.05 (∗∗∗∗: p ≤ 0.0001, ∗∗∗: p ≤ 0.001, ∗∗: p ≤ 0.01, ∗: p ≤ 0.05; ns indicates not significant). The detailed numbers of animals (n) used for the statistical tests in the individual experiments are shown in the respective figure legends.

## Supporting Citations

The following references appear in the supplemental information: Park et al.^59^; Su et al.^60^; Broquet et al.^61^
