# Supplementary material for: Clostridium butyricum-altered lung microbiome is associated with enhanced anti-influenza effects via G-protein-coupled receptor120
Source: iScience. 2025 Sep 4;28(10):113502. doi: 10.1016/j.isci.2025.113502 (PMC12483594; doi:10.1016/j.isci.2025.113502)
Supplement: Document S1. Figures S1–S6 and Table S1 [file mmc1.pdf]

**iScience, Volume *28***

## Supplemental information

# *Clostridium butyricum*-altered lung microbiome is associated with enhanced anti-influenza effects via G-protein-coupled receptor120

**Mao Hagihara, Makoto Yamashita, Tadashi Ariyoshi, Ayaka Minemura, Chika Yoshida, Seiya Higashi, Kentaro Oka, Motomichi Takahashi, Akinobu Ota, Akihiro Maenaka, Kenta Iwasaki, Jun Hirai, Yuichi Shibata, Takumi Umemura, Takeshi Mori, Hideo Kato, Nobuhiro Asai, and Hiroshige Mikamo**

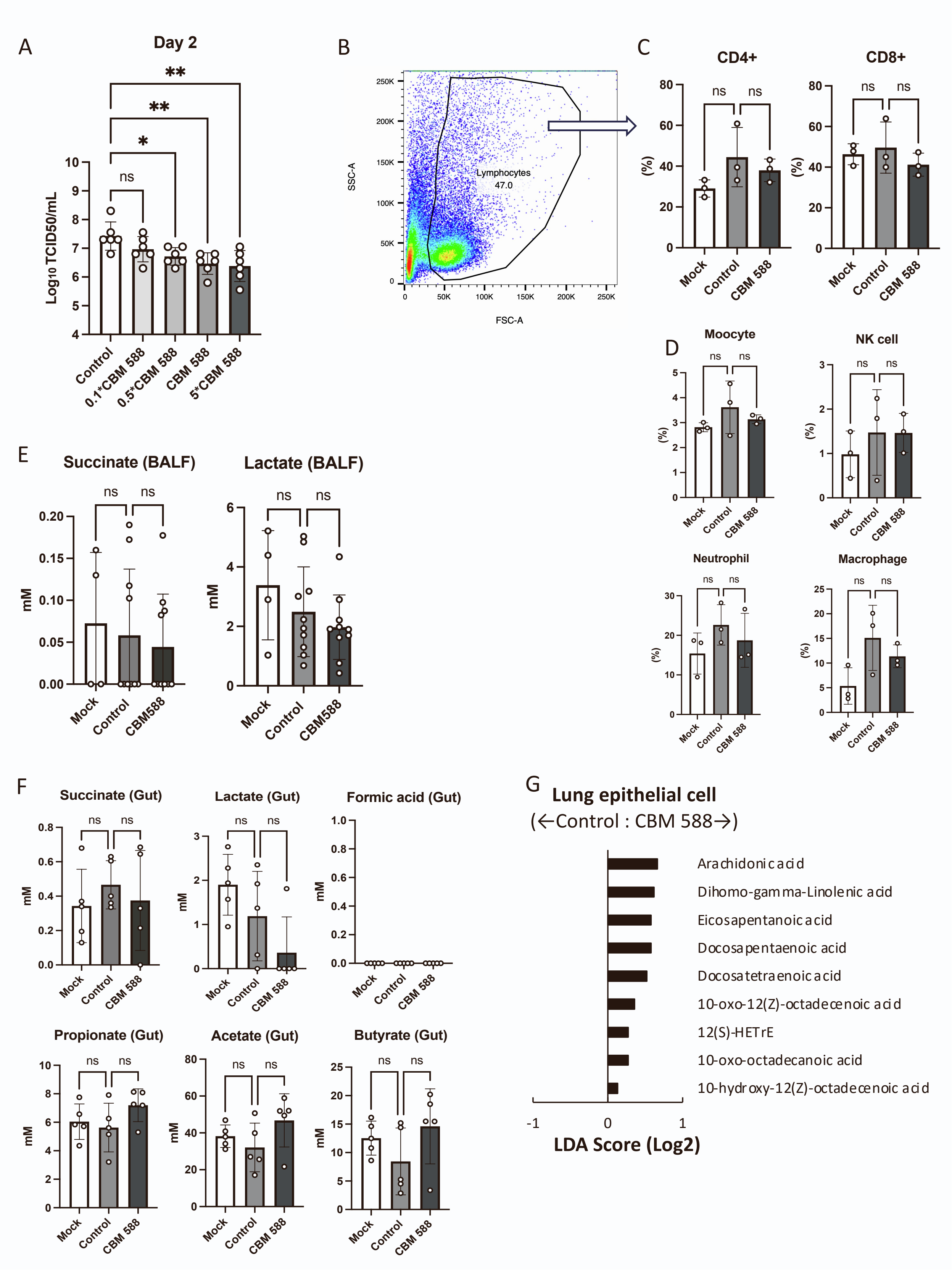


**Figure S1. CBM 588 demonstrates anti-influenza virus effects against influenza virus H3N2**

(A) Viral titers in the lungs on day 2 after influenza virus infection. All groups were infected with influenza A virus H3N2 (n = 6, respectively). The control group was treated with PBS. The other groups received orally administered CBM 588.

(B) Representative flow cytometry plots of lymphocyte expression in isolated cells. BALB/c mice infected with influenza virus A H3N2 received PBS (control) or *C. butyricum* (CBM 588) administrations for 16 d. Mock, n = 3; control, n = 3; and CBM 588, n = 3.

(C) Percentage of $CD4^+$ T cells ($CD3^+$ / $CD4^+$) and $CD8^+$ T cells ($CD3^+$ / $CD4^+$) in the lungs.

(D) Percentages of monocytes ($Ly6G^-$ / $7/4^+$ / $CD11b^+$), neutrophils ($Ly6G^+$ / $7/4^+$ / $CD11b^+$), NK cells ($NK1.1^+$ / $DX5^+$), and macrophages ($F4/80^+$ / $CD11b^+$) in the lungs.

(E) Short-chain fatty acid (SCFA) concentrations in BALF on day 2 post-infection.

(F) SCFA concentrations in fecal samples on day 2 post-infection.

(G) Linear discriminant analysis (LDA) score (Log2) of lipid metabolites shows significantly different peak areas between the control and CBM 588 groups in lung epithelial cells. Control, n = 6 and CBM 588, n = 6.

The results are presented as mean ± standard deviation (SD). Each dot represents an individual mouse. Results were considered statistically significant at $P < 0.05$. ns: not significant.

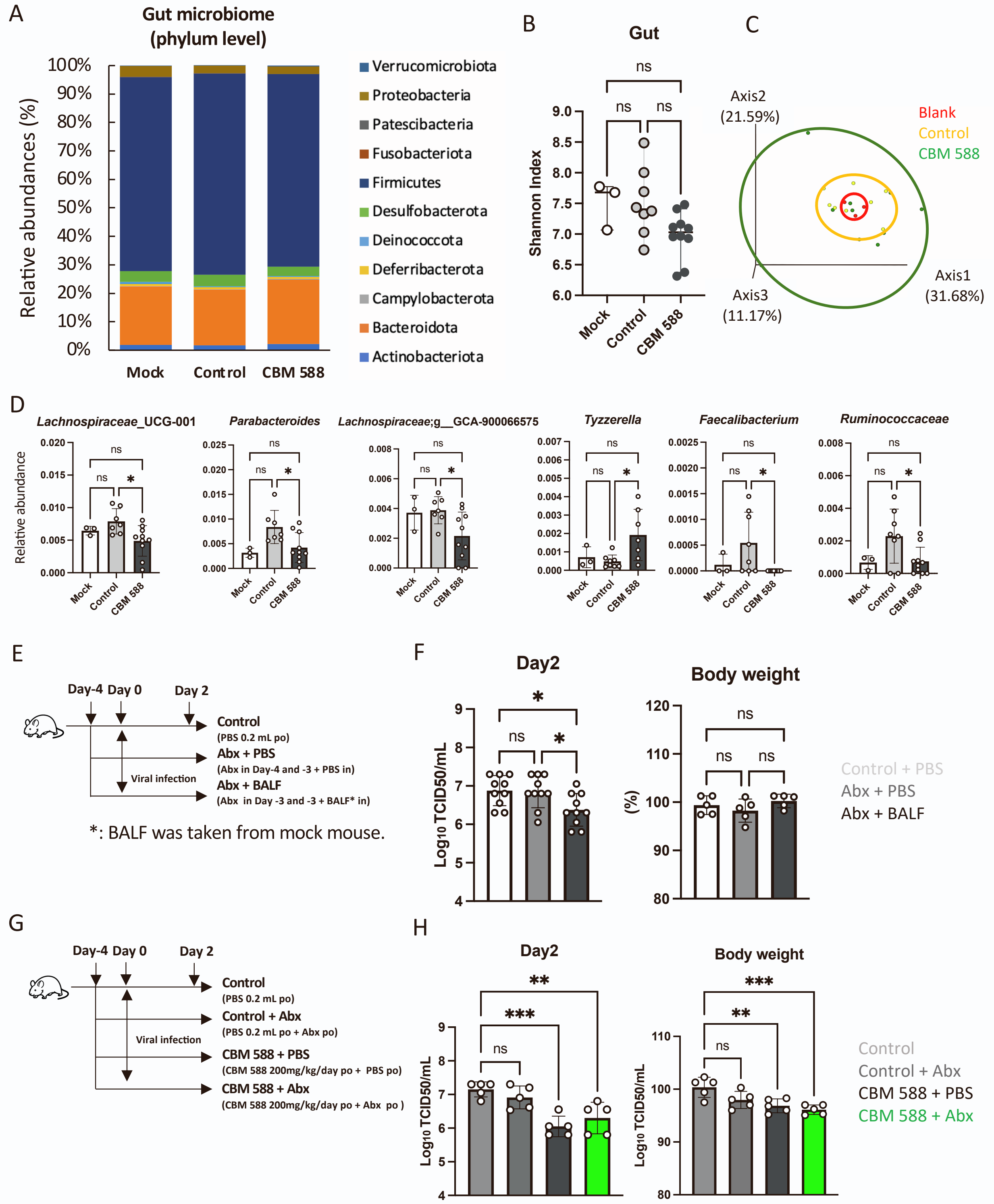


**Figure S2. Orally administered CBM 588 changes the gut microbiome**

(A) Bar graphs depict the mean relative abundance of bacterial families (> 1% relative abundance) in the gut microbiome at the phylum level for each group. BALB/c mice received PBS (mock), PBS (control), or *C. butyricum* (CBM 588) and were sacrificed 2 d after viral infection. The control and CBM 588 administered groups were infected with influenza virus H3N2. Mock, n = 4; control, n = 7; CBM 588 administration, n = 8.

(B) Comparison of the Shannon index among the different groups.

(C) Principal coordinate analysis (PCoA) was based on weighted UniFrac distances among the mock, control, and CBM 588 administration groups.

(D) Genus and species levels of relative species abundance (≥ 0.1%) in gut samples. Data are presented as the mean values of relative abundances ± SD.

(E) BALB/c mice were administered nasal PBS or an antibiotic mixture (Abx). All groups were infected with influenza A virus H3N2. The control group was treated with PBS. The other groups received Abx, PBS, Abx, or BALF. Control, n = 10; Abx + PBS, n = 10; Abx + BALF, n = 10.

(F) Viral titers in the lungs and body weight on day 2 after influenza viral infection.

(G) BALB/c mice were orally administered PBS, PBS and Abx, CBM 588, PBS, CBM 588 and Abx. All groups were infected with influenza A virus H3N2. Control, n = 5; Control + Abx, n = 5; CBM 588 + PBS, n = 5; CBM 588 + Abx, n = 5.

(H) Viral titers in the lungs and body weight on day 2 after influenza virus infection.

Each dot represents an individual mouse. Results were considered statistically significant when differences were $P < 0.05$ (***: $P \leq 0.001$, **: $P \leq 0.01$, *: $P \leq 0.05$; ns indicates not significant).

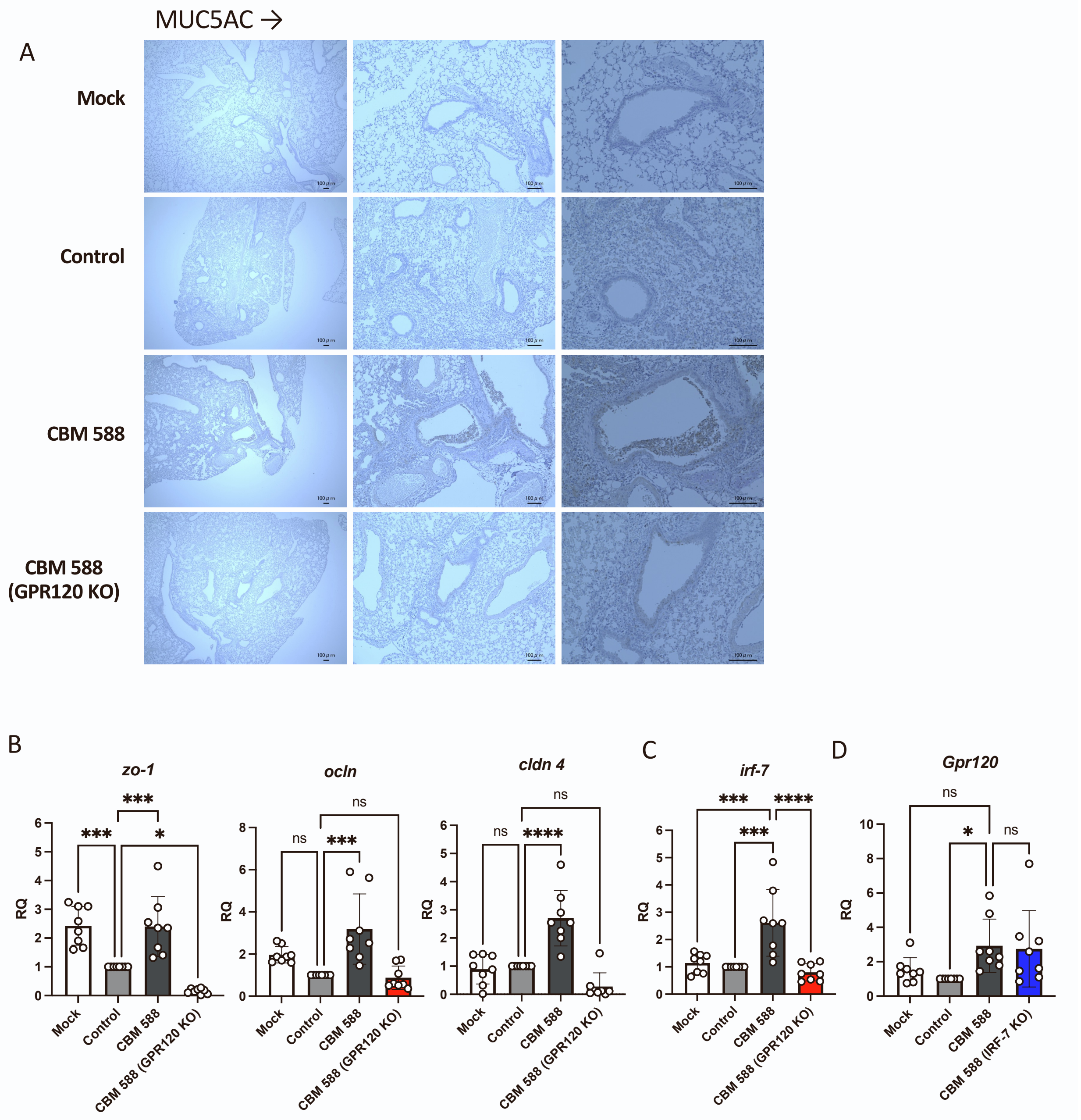


**Figure S3. GPR120 plays an important role in demonstrating the CBM 588-induced anti-influenza virus effects**

(A) Immunohistochemical analysis of MUC5AC expression in murine lungs. Representative lung histological images on day 2 after influenza virus infection (scale bar, 100 mm at the bottom right). Antibodies were incubated with Rabbit Anti-MUC5AC Polyclonal Antibody, unconjugated (bs-7166R), followed by conjugated secondary antibody, and 3,3'-diaminobenzidine (DAB) staining.

(B) Relative expression of tight junction proteins in the lung epithelial cells (RQ). BALB/c mice were administered PBS or *C. butyricum* (CBM 588) and sacrificed 2 days after viral infection. All groups were infected with the influenza virus H3N2, except for the mock group. Mock, n = 7; control, n = 7; CBM 588, n = 7.

(C) Relative expression of *Irf-7* genes in GPR120 KO mice in lung epithelial cells (RQ). Mice (wild-type or GPR120KO mice) were administered PBS or CBM 588 and were sacrificed 2 days after viral infection. All groups were infected with the influenza virus H3N2, except for the mock group. Mock, n = 7; Control, n = 7; CBM 588, n = 7; CBM 588 (GPR120 KO), n = 7.

(D) Relative expression of *Gpr120* genes in IRF-7 KO mice in lung epithelial cells (RQ). Mice (wild-type or IRF-7KO mice) received PBS or CBM 588 and were sacrificed two days after viral infection. All groups were infected with the influenza virus H3N2, except for the mock group. Mock, n = 7; Control, n = 7; CBM 588, n = 7; CBM 588 (GPR120 KO), n = 7.

The results are presented as mean ± standard deviation (SD). Each dot represents an individual mouse. Results were considered statistically significant when differences were $P < 0.05$ (****: $P \leq 0.0001$, ***: $P \leq 0.001$, **: $P \leq 0.01$, *: $P \leq 0.05$; ns indicates not significant).

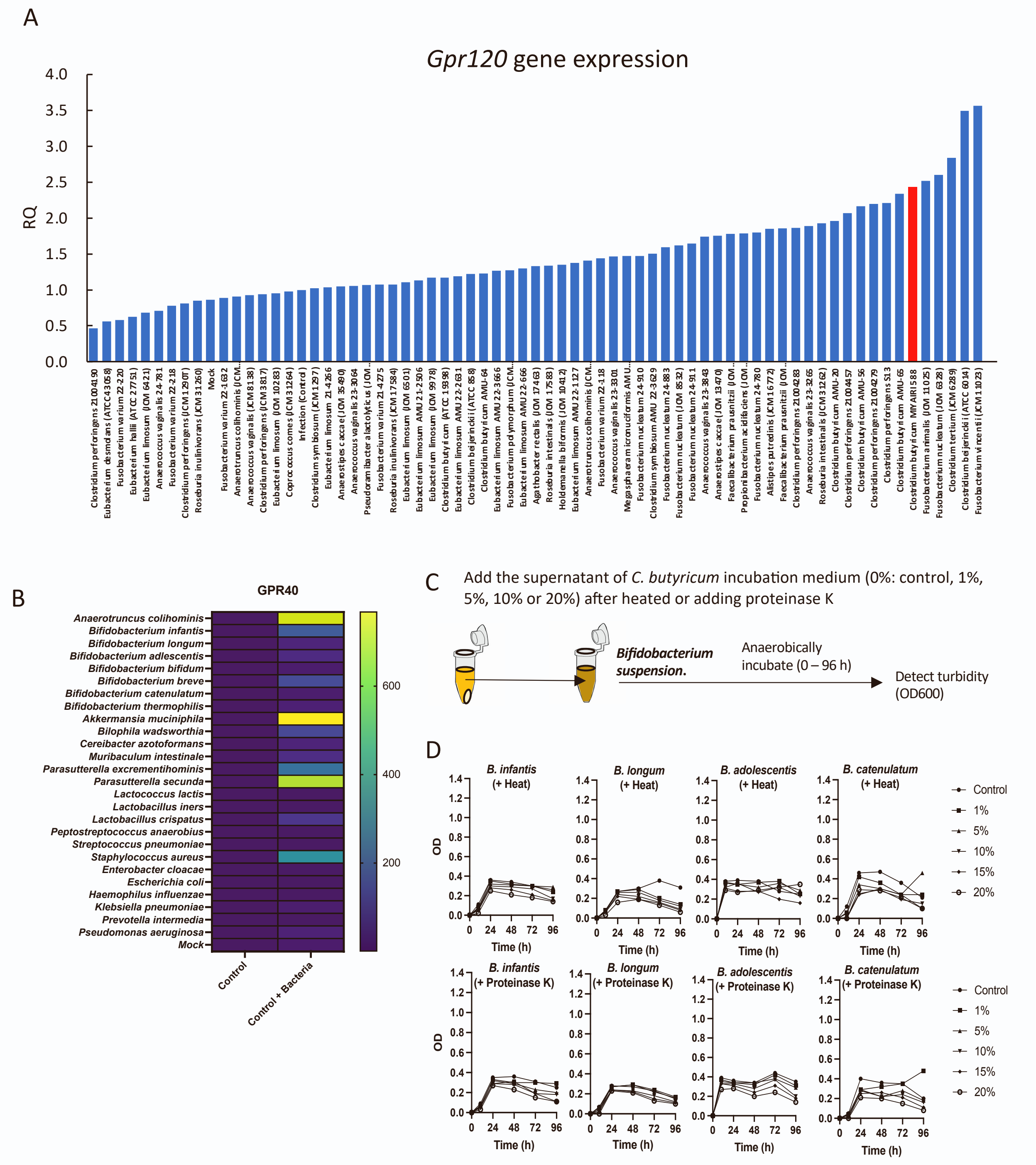


**Figure S4. CBM 588-induced *Bifidobacterium* species upregulates GPR120 expression**

(A) GPR120 expression in A549 (RQ) cells A549 cells were exposed to the supernatants of butyrate-producing bacterial media subjected to influenza virus H3N2 infection and incubated aerobically for 48 h, except for the mock group. Mock, n = 6; control, n = 6; butyrate-producing bacteria, n = 6.

(B) The heatmap represents the rate of GPR40 expression in A549 cells after 48 h of incubation. (A549 cells were exposed to the supernatants of each bacterial incubation medium subjected to influenza virus infection, whereas A549 cells were not exposed to bacteria under influenza virus infection).

(C) *Bifidobacterium* species concentrations during 0-96 h of incubation. Each *Bifidobacterium* species (n = 6, respectively). After heating or adding proteinase K, the supernatants of *C. butyricum* (CBM 588) incubation medium were added to *Bifidobacterium* species suspension at 0 (control), 1, 5, 10, 15, and 20%, and their turbidity was detected within 96 h of incubation.

(D) Turbidity of *the Bifidobacterium* species incubation medium. The results are presented as mean ± standard deviation (SD).

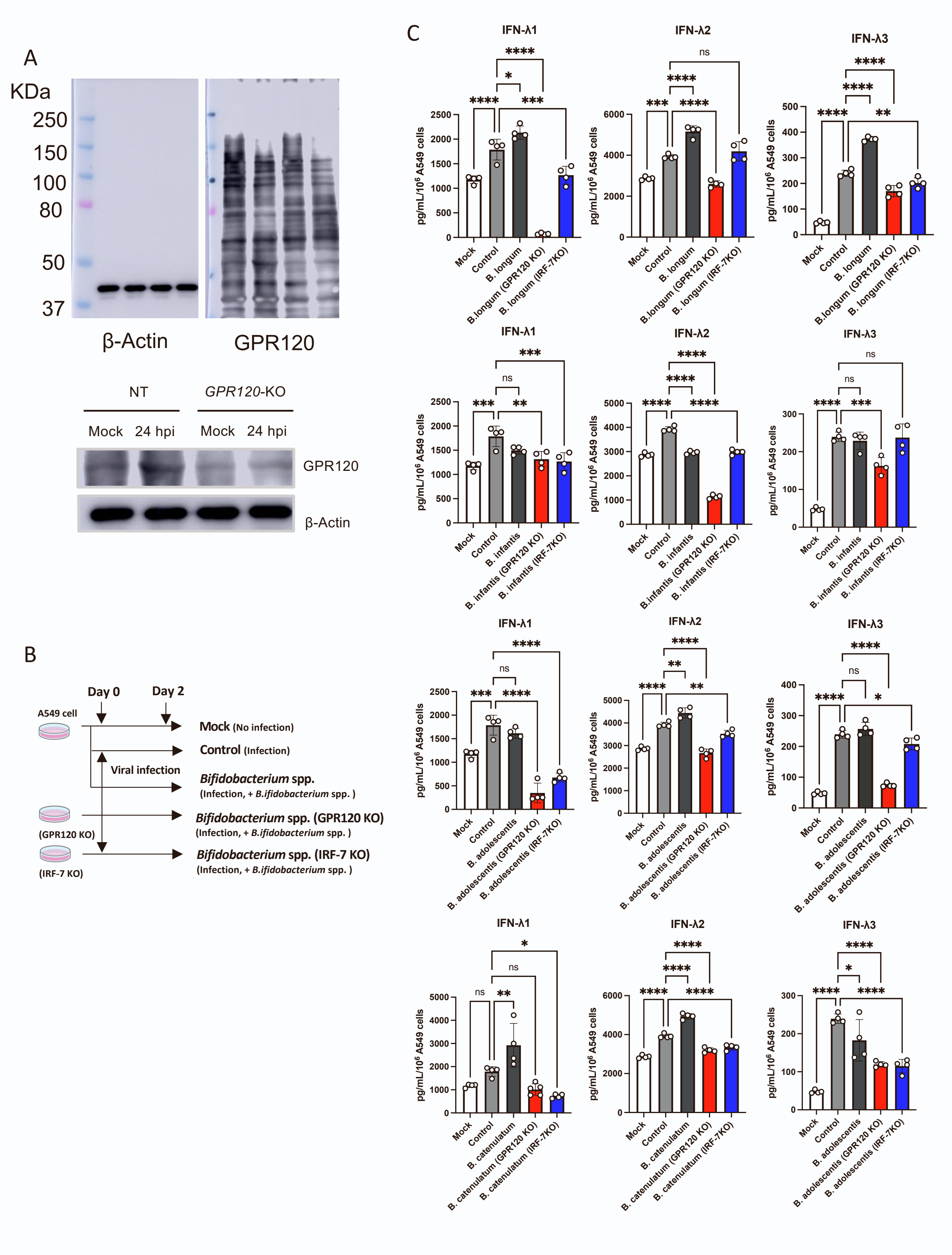


**Figure S5.** ***Bifidobacterium longum*** **promotes IFN-λ production through GPR120 upregulation**

(A) Confirmation of GPR120 expression suppression in A549 cells using western blotting. GPR120 expression knockout was performed using the CRISPR/Cas9 system.

(B) A549 cells (wild type, GPR120 KO, and IRF-7 KO) were exposed to *Bifidobacterium* species for 48 h under influenza virus H3N2 infection, except in the mock group.

(C) IFN-λ1, IFN-λ2, and IFN-λ3 expression levels. Mock, n = 6; control, n = 6; *Bifidobacterium* species, n = 6; *Bifidobacterium* species (GPR120 KO), n = 6; and *Bifidobacterium* species (IRF-7 KO).
The results are presented as mean ± standard deviation (SD). Each dot represents an individual mouse. Results were considered statistically significant when differences were $P < 0.05$ (****: $P \leq 0.0001$, ***: $P \leq 0.001$, **: $P \leq 0.01$, *: $P \leq 0.05$; ns indicates not significant).

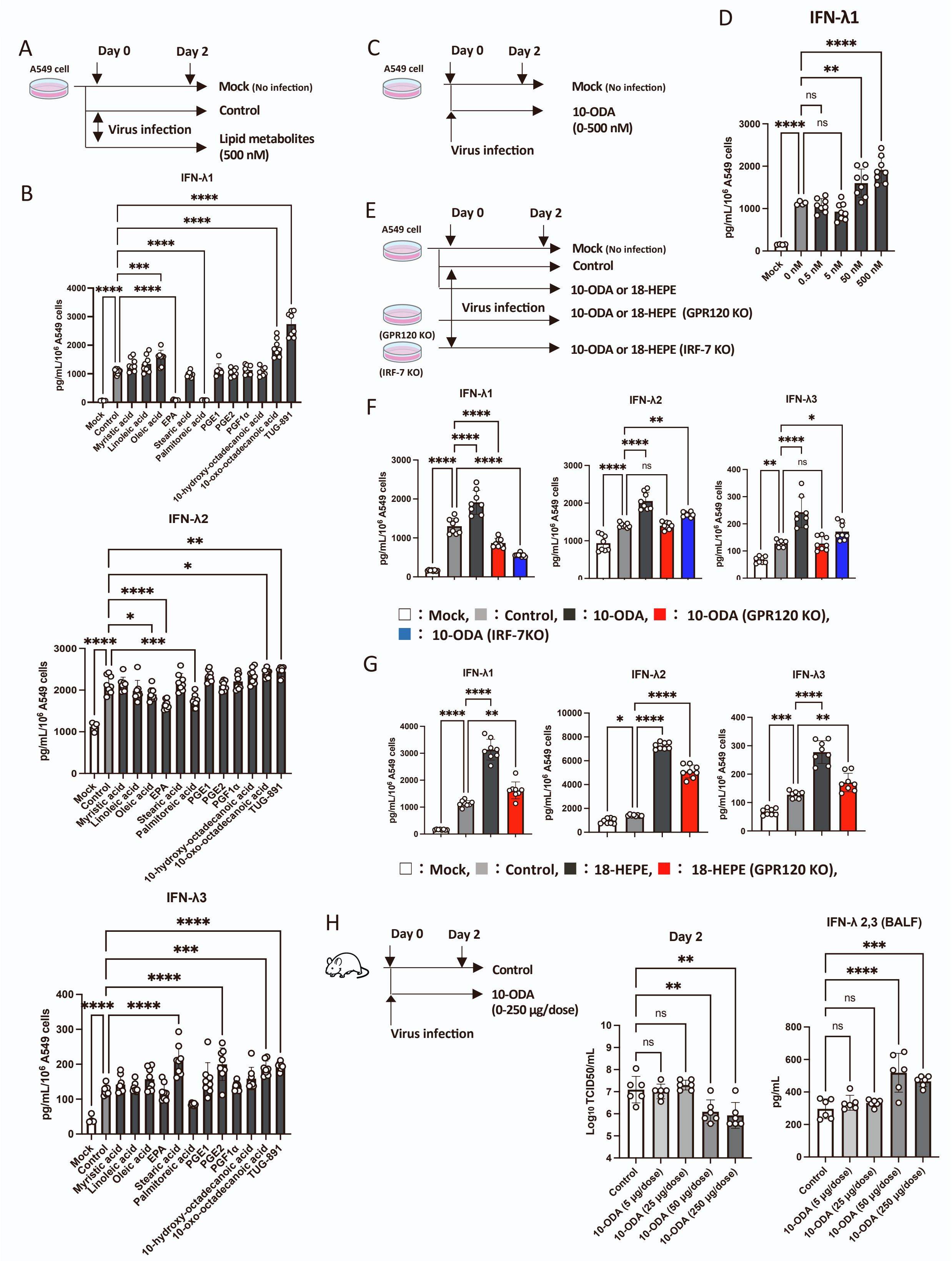


**Figure S6. *B. longum* complementally enhances LCFAs-induced anti-influenza virus effects through GPR120**

(A) A549 cell was exposed to long-chain fatty acids (LCFAs) at 500 nM for 48 h under influenza virus H3N2 infection, except for the mock group. Mock, n = 6; control, n = 6; each LCFAs, n = 6.
(B) IFN-λ1, IFN-λ2, and IFN-λ3 expression levels.
(C) A549 cells were exposed to 10-ODA (0-500 nM) for 48 h subjected to influenza virus H3N2 infection, except in the mock group.
(D) IFN-λ1 expression levels.
(E) A549 cells (wild-type, GPR120 KO, and IRF-7 KO) were exposed to 10-ODA or 18-HEPE at 500 nM for 48 h subjected to influenza virus H3N2 infection, except in the mock group. Mock, n = 8; control, n = 8; 10-ODA or 18-HEPE, n = 8; 10-ODA or 18-HEPE (GPR120 KO), n = 8; 10-ODA or 18-HEPE (IRF-7 KO); n = 8.
(F) IFN-λ1, IFN-λ2, and IFN-λ3 expression levels after 10-ODA treatment.
(G) IFN-λ1, IFN-λ2, and IFN-λ3 expression levels after 18-HEPE treatment.
(H) Viral titers in the lungs and body weight on day 2 after influenza viral infection. BALB/c mice were received 10-ODA treatments (0-250 μg/mouse), All groups were infected with influenza A virus H3N2. (n = 6, respectively).

The results are presented as mean ± standard deviation (SD). Each dot represents an individual mouse. Results were considered statistically significant when differences were $P < 0.05$ (****: $P \leq 0.0001$, ***: $P \leq 0.001$, **: $P \leq 0.01$, *: $P \leq 0.05$; ns indicates not significant).

**Table S1.** Primers used for quantitative real-time RT-PCR (mice)

| Name | Sequence<br>Forward (5'→3') | Reverse (5'→3') | Reference |
|---|---|---|---|
| ***irf-7*** | CACCCCCATCTTCGACTTCA | CCAAAACCCAGGTAGATGGTGTA | 70 |
| ***Gpr40*** | GGCCCTATAATGCCTCCAAT | CCAGGACCTGTTCCCAAGTA | 60 |
| ***Gpr120*** | GTGCCGGGACTGGTCATTGTG | TTGTTGGGACACTCGGATCTGG | 60 |
| ***Gpr41*** | GGGGTCGATACAAGAGT | CTGGCGGAGCTACGTGCT | 60 |
| ***Gpr43*** | CACGGCCTACATCCTCATCT | TTGGTAGGTACCAGCGGAAG | 61 |
| ***Gpr84*** | GACTGCCCCTCAAAAGACCTGC | GCCACGCCCCAGATAATTGC | 60 |
| ***Gpr109a*** | ATGGCGAGGCATATCTGTGTAGCA | TCCTGCCTGAGCAGAACAAGATGA | 60 |
| ***Ifn-λ2,3*** | AGCTGCAGGCCTTCAAAAAG | TGGGAGTGAATGTGGCTCAG | 71 |
| ***zo-1*** | ACTATGACCATCGCCTACGG | GGGGATGCTGATTCTCAAAA | 52 |
| ***ocln*** | CGGTACAGCAGCAATGGTAA | CTCCCCACCTGTCGTGTAGT | 52 |
| ***cldn4*** | GGGGATCATCCTGAGTTGTG | CACTGCATCTGACCTGTGCT | 52 |
| ***Ifn-λ1*** **(for A549)** | GCCATGGCTGCAGCTTGGAC | GGTGGACTCAGGGTGGGTTGAC | 72 |
| ***Ifn-λ2,3*** **(for A549)** | GACATGACCGGGGACTGCATG | GACACACAGGTCCCCGCTGG | 72 |
| ***Gapdh*** **(for A549)** | GTCTCCTCTGACTTCAACAGCG | ACCACCCTGTTGCTGTAGCCAA | 24 |
| ***β-actin*** **(for A549)** | CCCAGCCATGTACGTTGTA | AGGGCATACCCCTCGTAGATG | 72 |
| ***β-actin*** **(for mice sample)** | GTGCCGCCTGGAGAAACC | GGTGGAAGAGTGGGAGTTGC | 52 |
